# Power of Bayesian and Heuristic Tests to Detect Cross-Species Introgression with Reference to Gene Flow in the *Tamias quadrivittatus* Group of North American Chipmunks

**DOI:** 10.1093/sysbio/syac077

**Published:** 2022-12-12

**Authors:** Jiayi Ji, Donavan J Jackson, Adam D Leaché, Ziheng Yang

**Affiliations:** Department of Genetics, Evolution and Environment, University College London, London WC1E 6BT, UK; Department of Biology and Burke Museum of Natural History and Culture, University of Washington, Box 351800, Seattle, WA 98195-1800, USA; Department of Biology and Burke Museum of Natural History and Culture, University of Washington, Box 351800, Seattle, WA 98195-1800, USA; Department of Genetics, Evolution and Environment, University College London, London WC1E 6BT, UK

## Abstract

In the past two decades, genomic data have been widely used to detect historical gene flow between species in a variety of plants and animals. The *Tamias quadrivittatus* group of North America chipmunks, which originated through a series of rapid speciation events, are known to undergo massive amounts of mitochondrial introgression. Yet in a recent analysis of targeted nuclear loci from the group, no evidence for cross-species introgression was detected, indicating widespread cytonuclear discordance. The study used the heuristic method HYDE to detect gene flow, which may suffer from low power. Here we use the Bayesian method implemented in the program BPP to re-analyze these data. We develop a Bayesian test of introgression, calculating the Bayes factor via the Savage-Dickey density ratio using the Markov chain Monte Carlo (MCMC) sample under the model of introgression. We take a stepwise approach to constructing an introgression model by adding introgression events onto a well-supported binary species tree. The analysis detected robust evidence for multiple ancient introgression events affecting the nuclear genome, with introgression probabilities reaching 63%. We estimate population parameters and highlight the fact that species divergence times may be seriously underestimated if ancient cross-species gene flow is ignored in the analysis. We examine the assumptions and performance of HYDE and demonstrate that it lacks power if gene flow occurs between sister lineages or if the mode of gene flow does not match the assumed hybrid-speciation model with symmetrical population sizes. Our analyses highlight the power of likelihood-based inference of cross-species gene flow using genomic sequence data. [Bayesian test; BPP; chipmunks; introgression; MSci; multispecies coalescent; Savage-Dickey density ratio.]

## Introduction

Genomic sequence data are a rich source of information concerning the history of species divergences and cross-species gene flow. The past two decades have seen widespread use of genomic data to infer hybridization or introgression (Mallet *et al.*, 2016). Gene flow has been detected in a variety of species including Arabidopsis (Arnold *et al.*, 2016), butterflies (Martin *et al.*, 2013), corals (Mao *et al.*, 2018), lizards (Finger *et al.*, 2022), birds (Ellegren *et al.*, 2012), and mammals (Chan *et al.*, 2013; Kumar *et al.*, 2017; Shi and Yang, 2018). The studies have considerably enriched our understanding of the evolutionary dynamics of introgressed genes, and the role of introgression in speciation and ecological adaptation (Payseur and Rieseberg, 2016; Martin and Jiggins, 2017).

A number of statistical methods have been developed to analyze genomic sequence data to detect gene flow between species and to estimate its strength (as measured by the introgression probability or migration rate). Heuristic or summary methods are based on summaries of the multilocus sequence data and include the popular *D*-statistic or ABBA-BABA test (Patterson *et al.*, 2012), HYDE (Blischak *et al.*, 2018), and SNAQ (Solis-Lemus and Ane, 2016). The *D*-statistic and HYDE use the site-pattern counts for a species quartet to test for the presence of gene flow between nonsister species, while SNAQ uses the frequencies of estimated gene tree topologies. Likelihood methods use the multilocus sequence alignments directly and include the Bayesian implementations of the introgression model in PHYLONET/MCMC-SEQ (Wen and Nakhleh, 2018), *BEAST (Zhang *et al.*, 2018), and BPP (Flouri *et al.*, 2020), as well as the maximum-likelihood and Bayesian implementations of the continuous-migration model (also known as the isolation-with-migration or IM model) (Nielsen and Wakeley, 2001; Zhu and Yang, 2012; Dalquen *et al.*, 2017; Hey *et al.*, 2018). See Jiao *et al.* (2021) for a recent review. In theory, likelihood methods are expected to be more powerful because they use all information in the data about the model and parameters. However, summary and likelihood methods for inferring cross-species gene flow are seldom applied to the same real datasets with their utilities evaluated, partly because likelihood methods typically involve intensive computation and may not be computationally feasible for genome-scale datasets. In this regard, it is noteworthy that the BPP implementation of the multispecies-coalescent-with-introgression (MSci) model has been successfully applied to genomic datasets of more than 10,000 loci (Flouri *et al.*, 2020; Table 1; Thawornwattana *et al.*, 2022; Table S4).

**Table 1:** Summary of evidence for mitochondrial introgression in the *T. quadrivittatus* group (Sullivan *et al.*, 2014)

Species	Region	Distribution	Introgression	Source
*T. bulleri*	M	Allopatric	No	
*T. canipes* (C)	GB/RM	Allopatric	No	
*T. cinereicollis* (I)	GB/RM	Parapatric	Yes	Not assignable
*T. dorsalis* (D)	GB/RM	Parapatric	Yes	C/U/Q/Not assignable
*T. durangae*	M	Allopatric	No	
*T. palmeri*	GB/RM	Allopatric	Untested	
*T. quadrivittatus* (Q)	GB/RM	Parapatric	Yes	Not assignable
*T. rufus* (R)	GB/RM	Allopatric	No	
*T. umbrinus* (U)	GB/RM	Parapatric	Yes	Not assignable

*Note*: Geographic regions include Great Basin (GB), Rocky Mountains (RM), and Mexico (M). Single letter codes are for the six species included in the nuclear data analysis.

The *Tamias* chipmunks (*sensu lato*, but see Patterson and Norris, 2016) are a diverse group of at least 23 distinct species, occupying a variety of habitats in the western United States. Molecular phylogenetic studies have revealed a complex history of radiative speciations and cross-species gene flow involving morphologically and ecologically diverse lineages (Good and Sullivan, 2001; Good *et al.*, 2003).

**Figure 1.**
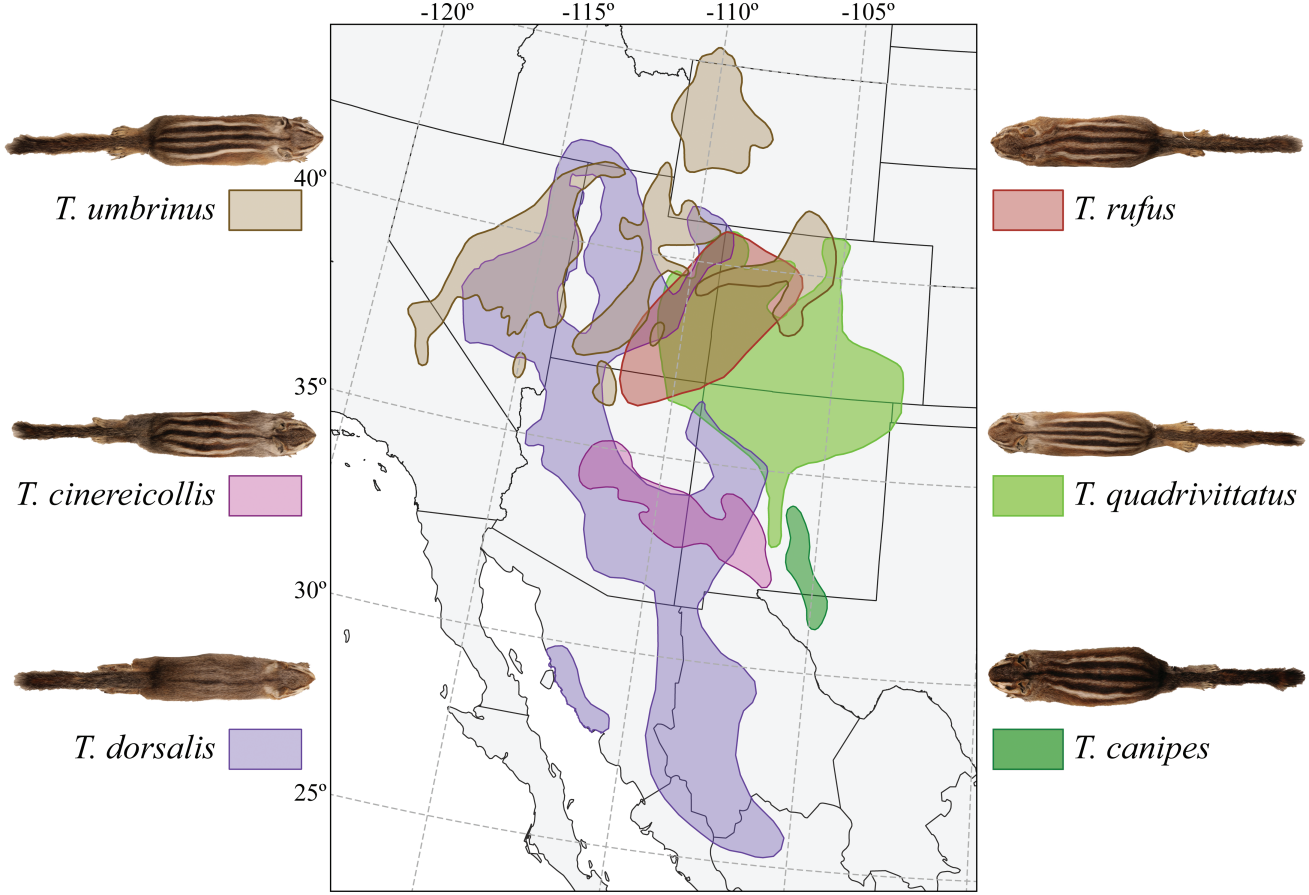
Geographic distributions of the six chipmunk species in the *Tamias quadrivittatus* group, based on data downloaded from the IUCN (https://www.iucnredlist.org/).

The *Tamias quadrivittatus* group of chipmunks currently consists of nine species that are distributed across the Great Basin along with the central and southern Rocky Mountains in North America (Fig. 1). Previous work on *Tamias* has highlighted the importance of genital morphology, specifically the baculum (a bone found in the penis) in male chipmunks, as a reliable indicator of species limits (Patterson and Thaeler Jr, 1982; White, 2010). The biogeographic history of the group likely included large range fluctuations that have periodically resulted in isolation and secondary contact among species, which would have affected opportunities for hybridization and/or introgression (Good *et al.*, 2003). The current distributions of species in the group have extensive regions of overlap and broad parapatry in ecological transition zones (Fig. 1), with instances of both allopatry and parapatry, and the determinants of current distributions are thought to be related primarily to competitive exclusion and ecological preference (Brown, 1971; Heller, 1971; Root *et al.*, 2001). The system provides an exciting opportunity to investigate the effects of introgression on genetic variation within and between species.

Hybridization between chipmunk species has been widely reported based on discrepancies between mtDNA, nuclear DNA, and morphology (Good and Sullivan, 2001; Good et al., 2003, 2008; Hird *et al.*, 2010). Work in the past decade has documented widespread mitochondrial introgression among species of the group (Reid *et al.*, 2012; Sullivan *et al.*, 2014; Sarver et al., 2017, 2021), which is often asymmetrical, possibly due to bacular morphology, which has been identified in at least six species (Good et al., 2003, 2008; Reid *et al.*, 2012; Sullivan *et al.*, 2014). Recent work on six species in the *T. quadrivittatus* group found that four of them exhibited clear evidence of introgressed mitochondrial DNA: *T. cinereicollis*, *T. dorsalis*, *T. quadrivittatus*, and *T. umbrinus* (Table 1). The cliff chipmunk (*T. dorsalis*) was involved in local introgression with multiple other species, receiving mtDNA from whichever congeneric chipmunk it came into contact with. However, populations of *T. dorsalis* that are geographically isolated carry mtDNA haplotypes that are unique to the species (Sullivan *et al.*, 2014; Sarver *et al.*, 2017). Range overlap in transition zones plays an important role in mitochondrial introgression in *Tamias* (Brown, 1971; Bi *et al.*, 2019).

Sarver *et al.* (2021) used a targeted sequence-capture approach to sequence thousands of nuclear loci (mostly genes or exons) to estimate the species phylogeny of the *T. quadrivittatus* group and to infer possible nuclear introgression. The program HYDE (Blischak *et al.*, 2018) was used to infer gene flow. Surprisingly, no significant evidence for gene flow involving the nuclear genome was detected between any species in the group, despite the evidence for widespread mitochondrial introgression. We note that HYDE, like the *D*-statistic, uses the four-taxon site-pattern counts pooled across the genome as data, and does not use information in the variation in genealogical history across the genome caused by the stochastic fluctuation of coalescent and introgression (Lohse and Frantz, 2014; Jiao *et al.*, 2021; Zhu and Yang, 2021). As a result, neither the *D*-statistic nor HYDE can detect gene flow between sister lineages. Importantly, HYDE is designed to estimate the relative genetic contributions of the two parental species which hybridized to form a third species. When applied to detect other modes of gene flow, it makes restrictive assumptions about the direction of gene flow, and about species divergence times and population sizes that may be unrealistic (Fig. 7). The performance of HYDE when its model assumptions are violated is unexplored.

To examine whether the lack of evidence for nuclear introgression in the analysis by Sarver *et al.* (2021) may be due to the lack of power of HYDE, here we re-analyze the data of Sarver *et al.* (2021) using the BPP program (Flouri et al., 2018, 2020), which includes a Bayesian implementation of the MSci model. Borrowing ideas from stepwise regression or Bayesian variable selection, we add introgression events sequentially onto the binary species tree to construct a joint MSci model with multiple introgression events. We develop a Bayesian test of introgression, calculating the Bayes factor for comparing the null model of no introgression against the alternative model of introgression via the Savage-Dickey density ratio (Dickey, 1971), using a Markov chain Monte Carlo (MCMC) sample under the MSci model. This may have a computational advantage over cross-model MCMC algorithms such as reversible jump MCMC (Green, 1995) or calculation of Bayes factors using thermodynamic integration (Gelman and Meng, 1998; Lartillot and Philippe, 2006). Our re-analysis revealed robust evidence for several ancient introgression events affecting the nuclear genome in the *Tamias* group, involving both sister and nonsister species. We examine the model assumptions underlying HYDE and use computer simulation to demonstrate that the opposite conclusions reached in the two analyses may be explained by the lack of power of HYDE to detect gene flow. We then assess the impact of ignoring introgression on estimation of population parameters, highlighting serious biases in species divergence time estimation when introgression exists and is ignored. Our results highlight the power of coalescent-based likelihood methods in the analysis of genomic datasets to infer the history of species divergence and gene flow.

## Theory: Bayesian Test of Introgression

### Bayes Factor Is Given by the Savage-Dickey Density Ratio in Comparisons of Nested Hypotheses

One can test for the presence of cross-species gene flow by comparing the introgression (MSci) model with the corresponding multispecies-coalescent (MSC) model with no gene flow. The model of no gene flow $\left(H_{0}\right)$ is a special case of the introgression model $\left(H_{1}\right)$, with $H_{1}$ reducing to $H_{0}$ when the introgression probability is 0.

The commonly used device for Bayesian model comparison is the Bayes factor, which is the ratio of the marginal likelihood values under the two compared models. When the two models are nested, the Bayes factor is given by the Savage-Dickey density ratio (Dickey, 1971). In general, suppose we wish to compare the null model $H_{0}:\phi =\phi_{0}$ against the alternative model $H_{1}:\phi \neq \phi_{0}$, and suppose that both models have common (nuisance) parameters λ, while parameters ξ in $H_{1}$ become unidentifiable when $\phi =\phi_{0}$. The parameter vector is λ for $H_{0}$ and (ϕ,λ,ξ) for $H_{1}$. Given data x, let the likelihood be $L_{0}\left(\lambda \right)$ under $H_{0}$ and L(ϕ,λ,ξ)=p(x | ϕ,λ,ξ) under $H_{1}$, with $L\left(\phi_{0},\lambda ,\xi \right)=L_{0}\left(\lambda \right)$ as the two models are nested. Let the prior be $\pi_{0}\left(\lambda \right)$ under $H_{0}$ and π(ϕ,λ,ξ)=π(ϕ)π(λ | ϕ)π(ξ | ϕ,λ) under $H_{1}$. The Bayes factor in support of $H_{1}$ over $H_{0}$ is defined as


$$\begin{array}{l} B_{10}=\frac{m}{m_{0}}=\frac{∭\pi (\phi ,\lambda ,\xi )L(\phi ,\lambda ,\xi )\;d\phi \;d\lambda \;d\xi }{\int \pi_{0}(\lambda )L_{0}(\lambda )d\lambda }, \end{array}$$ (1)

where $m_{0}$ and m are the marginal likelihoods for the two models, respectively.

Under the assumption that the priors on the common parameters (λ) agree between the two models


$$\begin{array}{l} \pi \left(\lambda \;|\;\phi_{0}\right)=\pi_{0}\left(\lambda \right), \end{array}$$ (2)

$B_{10}$ can be expressed as the ratio of the prior and posterior densities for ϕ in $H_{1}$, both evaluated at the null value $\phi_{0}$:


$$\begin{array}{l} B_{10}=\frac{m}{m_{0}}=\frac{\pi \left(\phi_{0}\right)}{\pi \left(\phi_{0}\;|\;x\right)}, \end{array}$$ (3)

where π(ϕ | x)=∬π(ϕ,λ,ξ | x)dξdλ is the marginal posterior density of ϕ.

A proof is provided in the Appendix. Note that equation (3) holds even when there exist nuisance parameters (λ) in both models, and also when the usual regularity conditions are not met: for example, when the null parameter values ($\phi_{0}$) are at the boundary of the parameter space in $H_{1}$, and when some parameters in $H_{1}$ (ξ) become unidentifiable when the parameters of interest take the null values (when $\phi =\phi_{0}$). Such nonstandard conditions cause considerable difficulties for the likelihood ratio test (LRT), leading to unconventional or unknown null distributions for the test statistic (Self and Liang, 1987). It is interesting that they do not cause any difficulty for the Bayesian test.

If the condition on the priors (equation (2)) does not hold, a correction factor may be applied (Verdinelli and Wasserman, 1995). This is not needed in our application.

### Calculation of the Savage-Dickey Density Ratio

The prior density $\pi \left(\phi_{0}\right)$ of equation (3) is typically available analytically. The posterior density $\pi \left(\phi_{0}\;|\;x\right)$ can be estimated using a kernel density smoothing procedure using the MCMC sample under $H_{1}$ (Silverman, 1986). This means that calculation of $B_{10}$ using equation (3) requires running the MCMC under $H_{1}$ only and no cross-model algorithms such as reverse-jump MCMC (Green, 1995) are needed. Note that within-model MCMC typically has better mixing properties than cross-model algorithms (Yang, 2014, pp. 247–260).

Suppose $\left(\phi^{\left(1\right)},\phi^{\left(2\right)},\cdots ,\phi^{\left(N\right)}\right)$ are an MCMC sample from the posterior π(ϕ | x). These are the ϕ values sampled during the MCMC, with the values for other parameters (λ and ξ) simply ignored. The kernel density estimator at the point $\phi_{0}$ is


$$\begin{array}{l} \hat{\pi }(\phi_{0}|x)=\frac{1}{Nh}\sum_{i=1}^{N}K\left(\frac{\phi_{0}-\phi^{\left(i\right)}}{h}\right), \end{array}$$ (4)

where K(⋅) is the kernel smoothing function and h is the smoothing parameter or window width. A good choice of h is (Silverman, 1986, eq. 3.30–3.31, p. 47)


$$\begin{array}{l} h=0.9\cdot min\left(SD,\frac{inter\text{-}quartile\;range}{1.34}\right)\times N^{-(1/5)}. \end{array}$$ (5)

The kernel function K is typically symmetrical around 0, with points further away from $\phi_{0}$ make less contribution to the density at $\phi_{0}$. For example, the Gaussian kernel is given as


$$\begin{array}{l} K(t)=\frac{1}{\sqrt{2\pi }}e^{-t^{2}/2}. \end{array}$$ (6)

**Figure 2.**
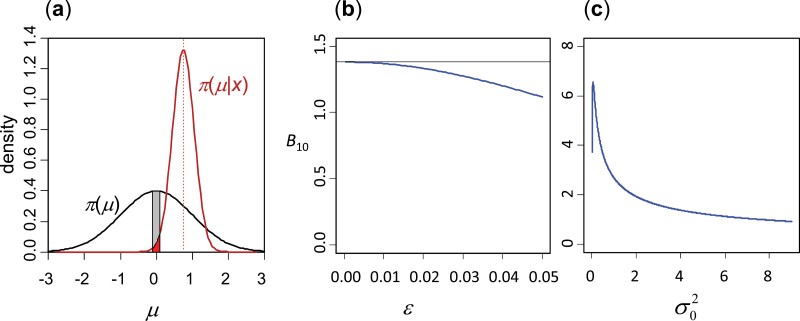
(a) Bayes factor expressed as the Savage-Dickey density ratio in the test of the null hypothesis $H_{0}:\mu =0$ against the alternative hypothesis $H_{1}:\mu \neq 0$, using a data sample from N(μ,1). The black and red curves represent the prior and posterior densities for μ in $H_{1}$, and the small interval (of width ε) in the parameter space for $H_{1}$ is the null interval *ϕ* (or interval of null effects), representing $H_{0}$. The prior and posterior probabilities over the null interval (the gray and red areas) depend on the interval width (ε), but when ε→0, their ratio converges to the Bayes factor $B_{10}=\pi \left(\mu_{0}\right)\;\;/\pi \left(\mu_{0}\;|\;x\right)$. If the area of null effects shrinks greatly when we move from the prior to the posterior, the data contain strong evidence against $H_{0}$. (b) Approximate Bayes factor $B_{10,\varepsilon }=P(\text{⧸}o)/P(\text{⧸}o\;|\;x)$ (equation (7)) plotted against ε for a dataset of size n=100 with the sample mean $\bar{x}=0.258$. The prior is $\mu ∼N(0,\sigma_{0}^{2})$ with $\sigma_{0}=2$ (twice the sampling standard deviation). When ε→0, $B_{10}=1.381$. (c) Bayes factor (equation (1) or (12)) plotted against the prior variance $\sigma_{0}^{2}$ for the same dataset showing the sensitivity of $B_{10}$ to the prior on the parameter of interest (μ). Note that in this dataset (with $\sqrt{n}|\bar{x}|=2.58$) $H_{0}$ is rejected by the LRT with p -value 1%.

However, this approach may be awkward to apply if the prior or posterior density at the null value, $\pi (\phi_{0})$ or $\pi (\phi_{0}\;|\;x)$, is 0 or ∞. In this paper, we use a more intuitive way of deriving the Savage-Dickey density ratio of equation (3), which also provides an approach to its calculation. This treats the problem of testing as a problem of estimation, and assesses how likely the parameter of interest (ϕ) differs from the null value ($\phi_{0}$). Define a null region or region of null effects, $\text{⧸}o:|\phi -\phi_{0}|<\varepsilon$, inside which ϕ is very close to $\phi_{0}$. The null region is a small part of the parameter space for $H_{1}$ that represents $H_{0}$ (Fig. 2). We then define a Bayes factor to represent the evidence for $H_{1}$


$$\begin{array}{l} B_{10,\varepsilon }=\frac{1-\mathbb{P}(\phi |x)}{\mathbb{P}(\phi |x)}/\frac{1-\mathbb{P}(\phi )}{\mathbb{P}(\phi )}\;\approx \frac{\mathbb{P}(\phi )}{\mathbb{P}(\phi |x)}, \end{array}$$ (7)

as 1−P(⧸o)≈1 and 1−P(⧸o|x)≈1 for small ε. When ε→0, $P(\text{⧸}o)\to \pi (\phi_{0})\Delta$ and $P(\text{⧸}o\;|\;x)\to \pi (\phi_{0}\;|\;x)\Delta$, where the differential Δ is the size of the null region, so that $B_{10,\varepsilon }\to \pi (\phi_{0})/\pi (\phi_{0}\;|\;x)$, as in equation (3). Thus the same conclusion is reached whether the problem is considered a testing problem (equation (1) or (3))) or an estimation problem (equation (7)).

The approach is illustrated in Fig. 2 using the simple problem of testing $H_{0}:\mu =0$ against $H_{1}:\mu \neq 0$ using a sample of size n from N(μ,1). The data are summarized as the sample mean $|\bar{x}|$. We assign the prior $\mu ∼N\left(0,\sigma_{0}^{2}\right)$ under $H_{1}$. The posterior is then $\mu |x∼N(\mu_{1},\sigma_{1}^{2})$, with $\mu_{1}=n\bar{x}/\;[\;\;\;(n+1)/\sigma_{0}^{2}]$ and $1/\sigma_{1}^{2}=n+(1/\sigma_{0}^{2})$. The prior and posterior probabilities of the null interval are $P(\text{⧸}o)=P\{|\mu |\;<\;\varepsilon \}=1-2\phi (-\varepsilon /\sigma_{0})\approx \pi (\mu_{0})\Delta$ and $P(\text{⧸}o\;|\;x)=\phi ([\varepsilon -\mu_{1}]/\sigma_{1})-\phi ([-\varepsilon -\mu_{1}]/\sigma_{1})\approx \pi (\mu_{0}\;|\;x)\Delta$, with the differential to be the width of the null interval, Δ=2ε.

The above-mentioned theory applies generally to Bayesian testing of nested hypotheses. Examples include comparison of different species delimitation models (e.g., one-species versus two-species models) (Yang and Rannala, 2010) and test of migration between species (e.g., two species with and without migration) (Nielsen and Wakeley, 2001). The theory may be applied to compare nonnested models as well if they both have the same null model as a special case. Suppose $H_{0}$ is a special case of both $H_{1}$ and $H_{2}$, and let $M_{0},M_{1},M_{2}$ be their marginal likelihood values. Then $B_{12}=M_{1}/M_{2}=B_{10}/B_{20}$, where the Bayes factors $B_{10}$ and $B_{20}$ can be calculated using the Savage-Dickey density ratio by running MCMC under $H_{1}$ and $H_{2}$. In practice, the approach has only limited precision and works only if the goodness of fit of the compared models is not drastically different. If both $H_{1}$ and $H_{2}$ fit the data much better than $H_{0}$ so that $B_{10}$ and $B_{20}$ are estimated to be ∞, no sensible estimate for $B_{12}$ can be generated.

### Test of Introgression

**Figure 3.**
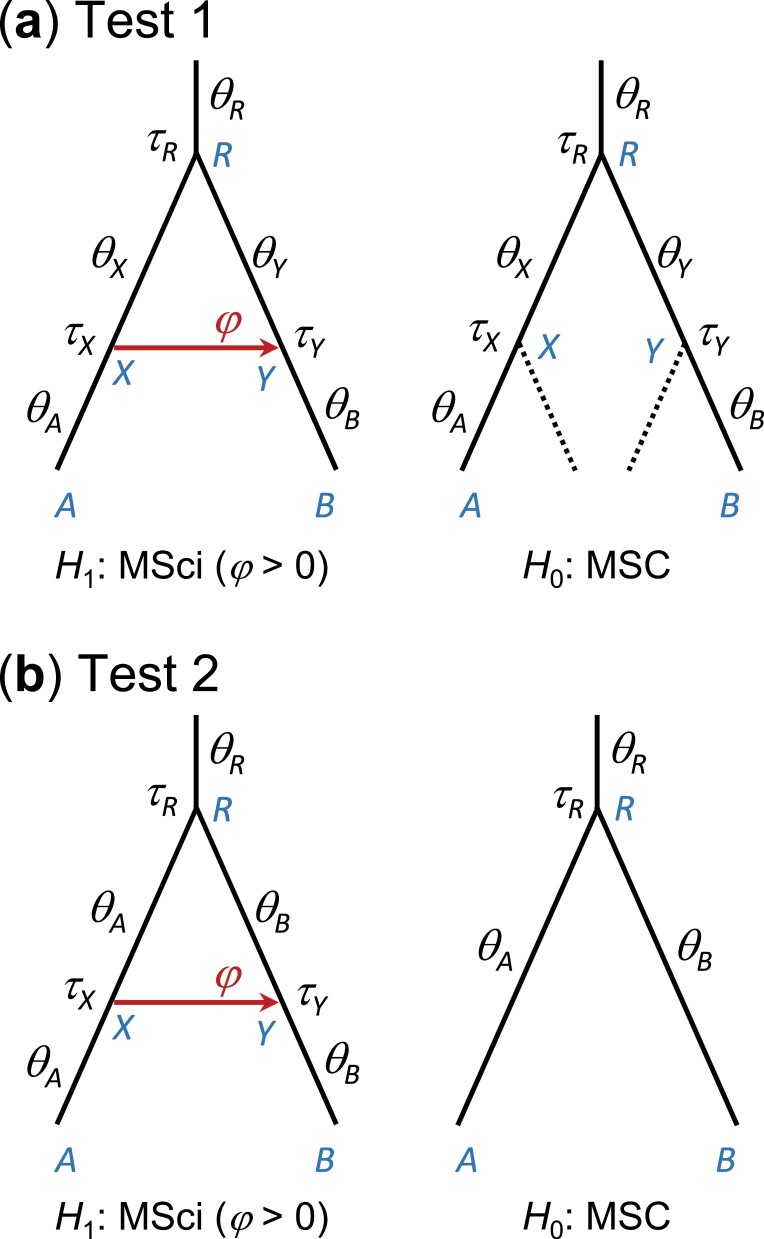
Parameters in the alternative and null hypotheses in two Bayesian tests of introgression (i.e., test of $H_{0}:\varphi =0$ against $H_{1}:\varphi >0$). The parameter of interest is the introgression probability φ. In test 1 (a), the shared parameters are $\lambda =\left(\tau_{R},\tau_{X}=\tau_{Y},\theta_{A},\theta_{B},\theta_{R},\theta_{X},\theta_{Y}\right)$. In test 2 (b), the shared parameters are $\lambda =\left(\tau_{R},\theta_{A},\theta_{B},\theta_{R}\right)$ while $\xi =\left(\tau_{X}=\tau_{Y}\right)$ in $H_{1}$ becomes unidentifiable at the null value $\varphi_{0}=0$. Here only the two species involved in introgression are shown. Including other species on the species tree adds the same set of parameters to the null and alternative hypotheses.

When we use the Savage-Dickey density ratio (equation (3)) to test introgression, the nuisance parameters include species divergence times (τ) and population sizes (θ) on the species tree. Since we use the same priors on τ and θ in models with and without introgresion, independent of the introgression probabilities (φ), the assumption of equation (2) holds. We consider two tests with different assumptions about the population size parameters (Fig. 3). In test 1, the MSci model assigns different θ parameters on the two segments of a branch broken by an introgression event; for example, in Fig. 3a branch RA is broken into two branches RX and XA and assigned $\theta_{X}$ and $\theta_{A}$, respectively. The null model of no gene flow will have two θ parameters for the branch as well. Such a model can be implemented in BPP by including ghost species in the MSC model from which no sequences are sampled (Fig. 3a). In the second test, the MSci model assigns the same θ parameter for a branch on the species tree before and after an introgression event (which can be specified using the control variable thetamodel = linked-msci in BPP) (Fig. 3b). When the introgression probability takes the null value (0) in $H_{1}$, the introgression time $\tau_{X}$ becomes unidentifiable. The proof of equation (A1) applies to both scenarios. In this study, we used test 1. Note that calculating the Bayes factor using the Savage-Dickey density ratio (equation (3) or (7)) requires an MCMC sample from $H_{1}$ and does not require any analysis or MCMC run under $H_{0}$.

In our BPP analysis, the introgression probability φ is assigned a beta prior beta(a,b), and the null hypothesis corresponds to $\varphi_{0}=0$ in $H_{1}$. Let the null region be ⧸o:φ<ε. Then P(⧸o)=P(φ<ε) in equation (7) is given by the cumulative distribution function (CDF) for beta(a,b), while P(⧸o | x) is simply the proportion of the sampled φ values that are <ε. Intuitively, the null region ⧸o:φ<ε in $H_{1}$ represents absence of introgression (as the introgression probability φ is negligibly small), [1−P(⧸o)]/P(⧸o) is the prior odds in favor of gene flow, while [1−P(⧸o | x)]/P(⧸o|x) is the posterior odds, and $B_{10}$ measures the change in the odds in favor of gene flow when we move from the prior to the posterior. We used ε=0.01 and confirm that use of ε=0.001 gave very similar results. A cutoff of 20 for $B_{10}$ may be considered strong evidence in support of $H_{1}$ (corresponding to 95% posterior for $H_{1}$ if the prior model probabilities for $H_{0}$ and $H_{1}$ are 1/2 each), while 100 means extremely strong evidence (corresponding to 99% posterior for $H_{1}$).

## Materials and Methods

### Chipmunk Genomic Data

The dataset, generated and analyzed by Sarver *et al.* (2021), includes 1060 nuclear loci from six chipmunk species: *T. rufus* (R), *T. canipes* (C), *T. cinereicollis* (I), *T. umbrinus* (U), *T. quadrivittatus* (Q), and *T. dorsalis* (D) (with 5, 5, 9, 10, 11, 11 individuals, respectively), as well as the outgroup *T. striatus* (3 individuals). We included all individuals whether or not their mtDNA was likely to be introgressed. Due to lack of a reference genome, Sarver *et al.* (2021) assembled genomic loci (targeted genes or exons) into contigs using an approach called Assembly by Reduced Complexity (ARC). Filters were then applied to remove missing data (contigs not present across all individuals) and sequences with likely assembly errors. The procedure generated a dataset of 1060 loci (1060 ARC contigs, Sarver *et al.*, 2021), with sequence length ranging from 14 to 1026 bp among loci and the number of variable sites from 0.33% to 15.2%.

High-quality heterozygous sites in the data, as identified by high mapping quality and depth of coverage, are represented using IUPAC ambiguity codes. They are accommodated using the analytical integration algorithm implemented in BPP (Gronau *et al.*, 2011; Flouri *et al.*, 2018). This takes the unphased genotype sequences as data and averages over all possible heterozygote phase resolutions, using their relative likelihoods based on the sequence alignment at the locus as weights (Huang *et al.*, 2022).

### Species Tree Estimation for the *T. quadrivittatus* Group

We used BPP version 4 (Rannala and Yang, 2017; Flouri *et al.*, 2018) to estimate the species tree under the MSC model without gene flow. This is the A01 analysis (speciesdelimitation = 0, speciestree = 1) (Yang, 2015).

**Figure 4.**
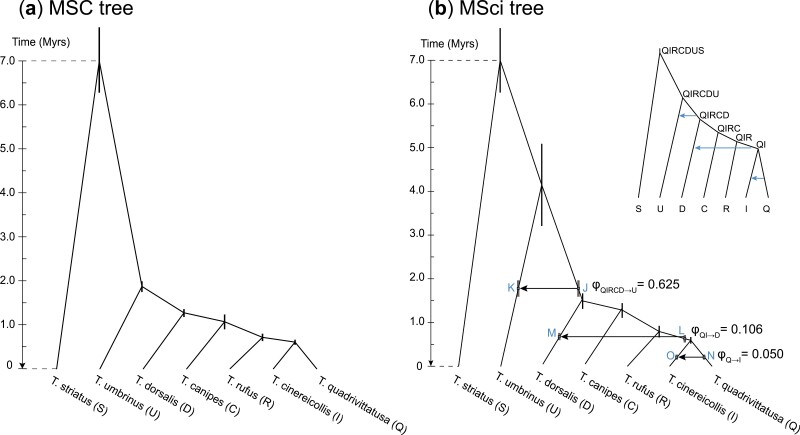
(a) Species tree for the *T. quadrivittatus* group with *T. striatus* used as the outgroup. Branch lengths represent the posterior means of divergence times (τ) estimated from BPP analysis of the full data of 1060 loci under the MSC model with no gene flow, with node bars indicating the 95% HPD intervals. A minimum divergence time of 7 Myrs for the outgroup *T. striatus* is used to convert the τ estimates into absolute times. (b) The joint introgression model constructed in this study with three unidirectional introgression events, showing parameter estimates from BPP analysis of the full data of 1060 loci. Nodes created by introgression events are labeled, with the labels used to identify parameters in Table S3. The MSci model includes 6 species divergence times and 3 introgression times (τ), 19 population size parameters (θ), and 3 introgression probabilities (φ).

We assigned inverse-gamma (IG) priors to parameters in the MSC model: θ∼ IG(3, 0.002) with mean 0.001 for population size parameters and $\tau_{0}∼$ IG(3, 0.01) with mean 0.005 for the age of the root. The shape parameter α=3 means that those priors are diffuse, while the prior means are based on estimates from preliminary runs. Note that both θ and τ are measured in the expected number of mutations per site. The inverse gamma is a conjugate prior for θ and allows the θ parameters to be integrated out analytically, leading to a reduction of parameter space and improved mixing of the MCMC algorithm. We conducted 10 replicate MCMC runs, using different starting species trees. Each run generated $2\times 10^{5}$ samples, with a sampling frequency of 2 iterations, after a burn-in of 16,000 iterations. Each run took about 70 hours using one thread on a server with Intel Xeon Gold 6154 3.0 GHz processors. Convergence was confirmed by consistency between runs. All runs converged to the same species tree (Fig. 4a), with ∼100%  posterior probability, which had the same topology as the tree inferred by Sarver *et al.* (2021).

### Stepwise Construction of the Introgression Model

As the species tree is well supported, apparently unaffected by cross-species introgression, we used the species tree to build an introgression model with multiple introgression events. Our procedure is similar to stepwise regression, the step-by-step method for constructing a regression model that involves adding or removing explanatory variables based on a criterion such as an F- or t-test.

Our procedure has two stages. In the first stage, we used BPP to fit a number of introgression models, each with only one introgression event, and rank candidate introgression events by their strength (indicated by the introgression probability φ). The analyses of Sarver *et al.* (2021) suggest that mitochondrial introgression affected mostly four species: *T. umbrinus* (U), *T. dorsalis* (D), *T. quadrivittatus* (Q), and *T. cinereicollis* (I). We considered introgression events involving all possible pairs among those four species, as well as another species, QI, the common ancestor of *T. cinereicollis* and *T. quadrivittatus* (Fig. 4a). The dataset of 1060 loci was analyzed under an MSci model with only one introgression event, estimating the introgression probability (φ) and introgression time (τ). We assign the same inverse-gamma priors on θ and τ as above, and beta(1,1) or U(0,1) for the introgression probability φ. Two replicate runs were conducted for each analysis to confirm consistency between runs, and MCMC samples from the two runs were then combined to produce posterior estimates of parameters. This analysis provides a ranking of the introgression events by the introgression probability. We calculated the Bayes factor for testing $H_{0}:\varphi_{0}=0$ given by the Savage-Dickey density ratio (equation (3)), using the null interval ⧸o=(0,0.01) (equation (7)); use of (0,0.001) produced virtually identical results. Only introgresssion events with $B_{10}\geq 20$ were considered further.

In the second stage, we added introgression events onto the binary species tree (Fig. 4a) sequentially in the order of decreasing strength (introgression probability). To reduce the computational cost and to examine the robustness of the analysis, this step was applied to two subsets of the 1060 loci: the first half and the second half, each of 530 loci. The priors used for population sizes and root age were as above. With multiple introgression events in the model, we extended the MCMC runs to be k-times as long if the model involved k introgression events. Three replicate runs were performed to check consistency between runs. Samples from the replicate runs were then combined to produce posterior summaries. At each step, the added introgression event was retained if it met the same cutoff as above in either of the two data subsets.

Our procedure produced a joint introgression model with three unidirectional introgression events. The joint model was then applied to the full dataset of 1060 loci to estimate the population parameters including introgression probabilities, introgression times, species divergence times, and population sizes (Fig. 4b), using the same prior settings. We conducted 3 replicate runs, using a burn-in of 50,000 iterations and then taking $10^{6}$ samples, sampling every 2 iterations. Each run took 200 hrs.

## Results

### Species Tree Estimation for the *T. quadrivittatus* Group

We analyzed the full data of 1060 loci under the MSC model without gene flow to estimate the species tree. The ten replicate runs using different starting species trees converged to the same maximum *a posteriori* probability (MAP) tree, with posterior probability ∼100%  (Fig. 4a). Sarver *et al.* (2021) recovered the same species tree topology in their analysis of the same data using ASTRAL (Mirarab and Warnow, 2015) and SVDQUARTETS (Chifman and Kubatko, 2014), although with weaker support for some nodes, e.g., concerning the placement of *T. rufus*. The differences in support may be due to the fact that ASTRAL and SVDQUARTETS use summaries of the multilocus sequence data that are not sufficient statistics, and are thus less efficient than the full likelihood method implemented in BPP (Xu and Yang, 2016; Zhu and Yang, 2021).

### Stepwise Construction of the Introgression Model

In the first stage of our procedure, we fitted introgression models, each involving one introgression event, using the full dataset of 1060 loci. We considered introgression events between every contemporary pair of the five species: *T. cinereicollis* (I), *T. dorsalis* (D), *T. quadrivittatus* (Q), and *T. umbrinus* (U), and the ancestral species QI (Fig. 4a). Introgression events that passed our cutoff ($B_{10}\geq 20$) are listed in Table 2. Introgression from QI into D had the highest probability, >10% , while six more events had φ>5% : Q → D, D → QI, QI → U, I → D, Q → I, and I → Q. We note that introgressions between Q and I, and between QI and D, were significant in both directions and the estimated introgressions times were close (Table 2). We thus replaced the two unidirectional introgression events by one bidirectional introgression in further analyses (model D in Flouri *et al.*, 2020).

**Table 2:** Posterior means and 95% HPD CIs (in parentheses) for introgression probability (φ) and introgression time (τ) in the separate introgression analysis

	Introgression	φ	τ ($10^{-3}$)	$B_{10}$
*	QIRCD → U	0.6215 (0.3907, 0.8243)	0.896 (0.784, 1.004)	∞
*	QI → D	0.1187 (0.0866, 0.1499)	0.337 (0.311, 0.367)	∞
	Q → D	0.0779 (0.0509, 0.1026)	0.297 (0.253, 0.328)	∞
	D → QI	0.0707 (0.0384, 0.1058)	0.337 (0.302, 0.366)	∞
	QI → U	0.0624 (0.0269, 0.1020)	0.408 (0.353, 0.457)	21.27
	I → D	0.0579 (0.0332, 0.0862)	0.265 (0.217, 0.318)	∞
*	Q → I	0.0568 (0.0315, 0.0750)	0.098 (0.073, 0.121)	∞
	I → Q	0.0533 (0.0153, 0.0969)	0.111 (0.077, 0.156)	∞
	D → U	0.0214 (0.0022, 0.0483)	0.276 (0.178, 0.474)	0.04
	Q → U	0.0198 (0.0037, 0.0389)	0.296 (0.209, 0.367)	0.05
	D → I	0.0180 (0.0092, 0.0275)	0.155 (0.123, 0.192)	0.39
	D → Q	0.0177 (0.0058, 0.0315)	0.184 (0.117, 0.347)	0.10
	U → QI	0.0097 (0.0022, 0.0181)	0.371 (0.322, 0.410)	0.01
	I → U	0.0069 (0.0015, 0.0136)	0.158 (0.098, 0.223)	0.00
	U → D	0.0066 (0.0024, 0.0112)	0.235 (0.176, 0.300)	0.00
	U → Q	0.0061 (0.0008, 0.0127)	0.200 (0.119, 0.294)	0.00
	U → I	0.0037 (0.0009, 0.0071)	0.147 (0.090, 0.207)	0.00

*Note*: The species tree of Fig. 4a is used, with a single introgression event assumed in each analysis. The full dataset of 1060 loci is analyzed using bpp to estimate the introgression probability (φ) and the introgression time (τ), together with the species divergence times (τ) and population sizes (θ) on the species tree. Introgression events with $B_{10}<$ 20 (D → U and below) are not considered further in the stepwise approach of constructing the joint introgression model. The three introgression events that are selected in the joint introgression model are marked with asterisks. Bayes factor $B_{10}=\infty$ occurs if all φ values in the MCMC sample are >ε=1% .

The time of QI → U introgression was estimated to be 0.000408, very close to the species divergence time at node QIR (0.000417) (Fig. 4a), suggesting that the introgression was probably a more ancient event. Note that if an introgression event is assigned incorrectly to a daughter branch to the lineage truly involved in introgression, one would expect the estimated introgression time to collapse onto the species divergence time. We thus attempted to place the introgression onto more ancient ancestral branches on the species tree (Fig. 4a) and finally identified the lineage involved in introgression to be the ancestral species QIRCD. The QIRCD → U introgression had an estimated time that was away from the species divergence times, and the estimated introgression probability (62%) was the highest (Table 2).

In the second stage, we added introgression events identified in Table 2 onto the binary species tree of Fig. 4a, in the order of their introgression probabilities (Table S1). This was applied to two data subsets (the full data split into two halves). While our procedure allows introgression events already in the model to drop out when new introgressions are added to the model, this did not happen in the analysis of the *Tamias* dataset. Instead the most important introgression events identified in stage 1 remained to be most important in the joint introgression models constructed in stage 2. Note that multiple introgression events may not be independent. An introgression event significant in stage 1 may not be significant anymore when other introgression events are already included in the model. For example, when the QI → D introgression was already included in the model, none of the introgressions Q → D, D → QI, I → D and I → Q was significant. Those introgressions may be expected to lead to similar features in the sequence data, such as reduced sequence divergences between Q or I and D. Similarly, introgression probability for an introgression event often became smaller when other introgressions were added in the model. However, the opposite may occur as well. For example, $\varphi_{QIRCD\to U}$ was estimated to be 54-63% when this was the only introgression assumed in the model, but increased to 59-69% when other introgression events were added in the model (Table S1).

Results for the two data subsets were largely consistent, especially concerning introgression events with high introgression probabilities. We thus arrived at a joint introgression model with three unidirectional introgression events (Fig. 4b, Table S1).

We examined the impact of the prior for φ on the Bayesian test of introgression. We calculated the Bayes factor $B_{10}$ using the full dataset of 1060 loci under the prior φ∼ beta(α,β), with α=0.2,1,5 and β=0.2,1,5, generating nine prior settings (Table S2). Note that beta(α,β) has the mean E(φ)=α/(α+β) and variance $V\left(\varphi \right)=\alpha \beta /({\left(\alpha +\beta \right)}^{2}\left(\alpha +\beta +1\right))$. In particular, the prior mean varied from 0.0385 for beta(0.2, 5) to 0.961 for beta(5, 0.2). The Bayes factor $B_{10}$ was ∞ for all three introgression probabilities in the joint model, insensitive to the prior on φ (Table S2).

### Estimation of Introgression Probabilities and Species Divergence/Introgression Times

Finally, we fitted the joint introgression model of Fig. 4b to the full data of 1060 loci, as well as the two halves, with parameter estimates shown in Table S3. The fitted model is very parameter-rich, partly as we assign different θ parameters for different branches on the species tree: for example, branch Q in Figure 4b is broken into two segments by the introgression event, Q → I, which are assigned two independent θ parameters. As a result, population sizes for ancestral species tend to be poorly estimated, especially for those populations with a very short time duration. These patterns are consistent with simulation studies that examine the information content in multilocus datasets (Huang *et al.*, 2020).

The estimated introgression probabilities from the full data are 0.625 with the 95% highest probability density (HPD) credibility interval (CI) to be (0.442, 0.794) for $\varphi_{QIRCD\to U}$, 0.106 (0.074, 0.139) for $\varphi_{QI\to D}$, and 0.050 (0.028, 0.074) for $\varphi_{Q\to I}$. The introgression probability $\varphi_{QIRCD\to U}$ involved considerable uncertainty, with a large CI, possibly because the introgression is ancient and is between sister species, making it hard to estimate its strength, so that the dataset of 1060 loci may be too small.

**Figure 5.**
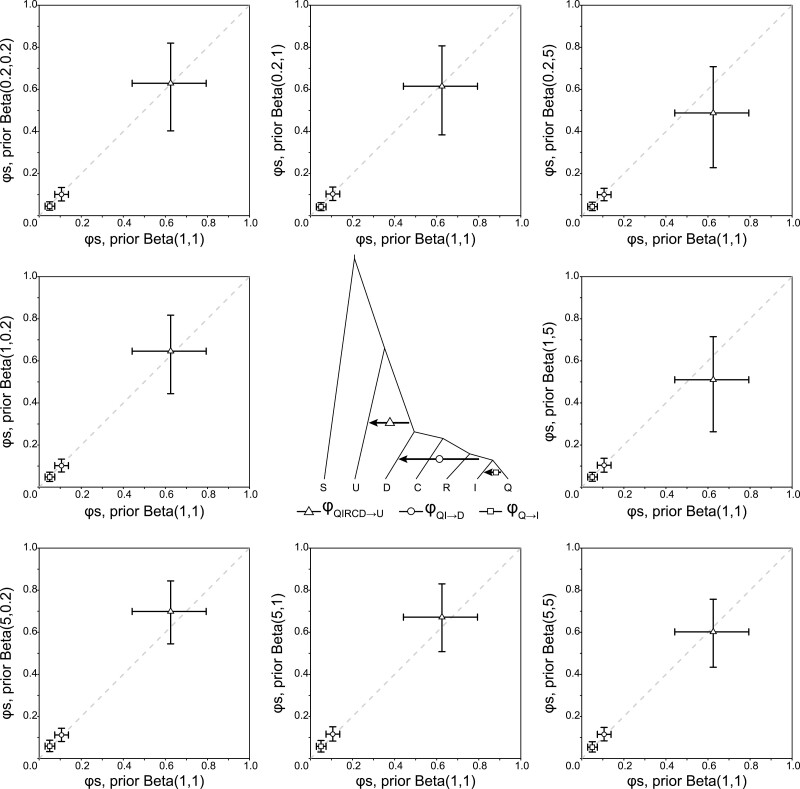
Posterior means and 95% HPD CIs for the three introgression probabilities (φ) obtained from BPP analyses of the full data of 1060 loci using different beta priors, φ∼ beta(α,β).

We evaluated the impact of the prior for φ on parameter estimation in the analysis of the full dataset, using α=0.2,1,5 and β=0.2,1,5 in the prior φ∼ beta(α,β) (Fig. 5). The prior had some effects on $\varphi_{QIRCD\to U}$, with the prior mean being more important than the prior variance. Under beta(0.2, 5) with the prior mean 0.0385, the posterior mean was lower, and the CI wider. Under beta(5, 0.2) with the prior mean 0.961, the posterior mean was higher, and the CI narrower. However, the posterior CIs overlapped considerably among the different priors, and overall the impact of the prior for φ on the estimate of $\varphi_{QIRCD\to U}$ was minor. Estimates of $\varphi_{QI\to D}$ and $\varphi_{Q\to I}$ were insensitive to the prior used (Fig. 5).

**Figure 6.**
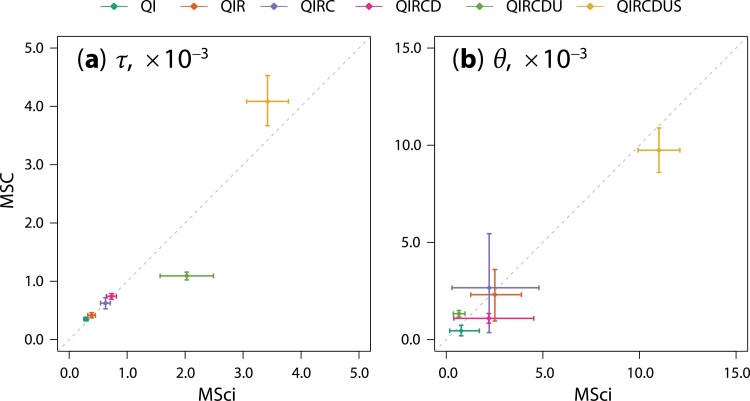
Scatterplot of posterior means and 95% HPD CIs (a) for the six species divergence times (τ) and (b) for the six ancestral population sizes (θ) in the MSC and MSci models of Fig. 4 obtained from BPP analyses of the full data of 1060 loci. Note that both τ and θ are measured in the expected number of mutations per site.

Accommodating gene flow in the model had significant impacts on estimation of the time of divergence between species involved in gene flow (Figs. 4 and 6). While estimates of times for the recent divergences ($\tau_{QI},\tau_{QIR},\tau_{QIRC}$, and $\tau_{QIRCD}$) were nearly identical between the MSC model ignoring gene flow and the MSci model incorporating gene flow, the estimated age of the *T. quadrivittatus* clade ($\tau_{QIRCDU}$) was much greater under MSci than under MSC (Fig. 6). This can be explained by the fact that the MSC model ignored the QIRCD → U introgression, which had introgression probability 62.5%. Note that sequence divergence between any pair of species X and Y has to be older than species divergence ($t_{XY}>\tau_{XY}$), and as a result, the minimum (rather than average) sequence divergence dominates the estimate of species divergence time. If gene flow is present between species and is ignored in the model, the reduced sequence divergence due to gene flow will be misinterpreted as recent species divergence, leading to underestimation of species divergence time. This effect has been noted in previous simulations (Leaché *et al.*, 2014).

The estimated age of the root of the species tree ($\tau_{QIRCDUS}$) was slightly smaller under MSci than under MSC. However, $\tau_{QIRCDUS}$ is negatively correlated with the population size ($\theta_{QIRCDUS}$) so that both parameters have large uncertainties (Burgess and Yang, 2008).

Sullivan *et al.* (2014, Fig. 1) used the minimum divergence time of 7 Ma for the outgroup species *T. striatus*, based on fossil teeth thought to belong to *Tamias* found in the late Miocene, reported by Dalquest *et al.* (1996), to date the *T. quadrivittatus* clade to 1.8 Ma in a maximum-likelihood concatenation analysis of four nuclear genes, and to 1.2 Ma (with 95% CI 0.6–2.2) in a *BEAST (Heled and Drummond, 2010) analysis of the same data. Concatenation analysis is known to be biased as it does not accommodate the stochastic variation of gene tree topologies and divergence times among loci due to the coalescent process (Ogilvie *et al.*, 2017). We used the same calibration to rescale the estimates of τ under the MSC and MSci models (Fig. 4). The minimum age for the *T. quadrivittatus* clade was 1.9 Ma (with 95% HPD CI to be 1.8–2.0) under the MSC model, comparable to the *BEAST estimate under the same model (Fig. 4a). Under the MSci model, the estimated minimum age was 4.1 Ma (with CI be 3.2–5.1) (Fig. 4b), much older than the estimates under the MSC model without gene flow. Note that here the CIs accommodate the uncertainty due to finite amounts of sequence data but not uncertainties in the fossil calibration.

### Model Assumptions Underlying HYDE

Whereas the analyses of nuclear data by Sarver *et al.* (2021) using HYDE detected no significant signal of introgression at all, our BPP analyses of the same data revealed strong evidence of multiple introgression events, involving both sister and nonsister species (Fig. 4b). To understand the opposing conclusions reached in the two analyses, here we examine the model assumptions underlying HYDE. We then use simulation to compare the performance of HYDE and BPP under conditions that are representative of the *Tamias* data but may violate the assumptions of HYDE.

**Figure 7.**
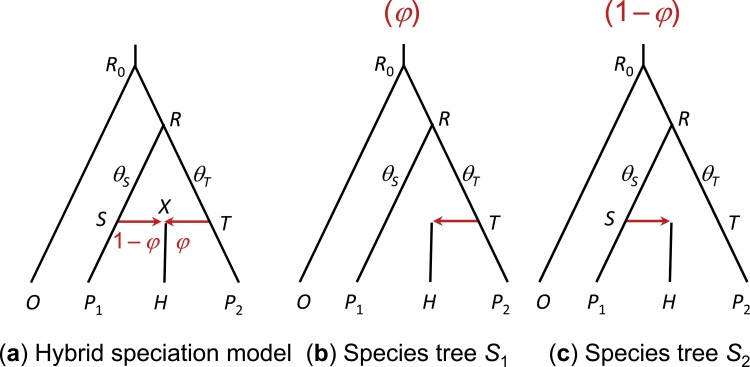
(a) HYDE assumes a hybrid-speciation model with the additional assumption of equal population sizes, or a symmetrical inflow model, with $\tau_{S}=\tau_{T}$ and $\theta_{S}=\theta_{T}$ (Blischak *et al.*, 2018). (b, c) Two parental species trees $S_{1}$ and $S_{2}$ induced by the hybridization model of (a). Site patterns are a mixture over the two species trees.

HYDE was developed under the hybrid-speciation model of Fig. 7a, with $\tau_{S}=\tau_{X}=\tau_{T}$, and $\theta_{S}=\theta_{T}$ (Blischak *et al.*, 2018). Formulated for quartet data, with one sequence from each of the four species, it uses the counts or frequencies of three parsimony-informative site patterns: iijj, ijji, ijij, to estimate the genetic contributions of the two parental species to the hybrid species: φ and 1−φ. Here pattern ijkl means a site with nucleotides i,j,k,l in $O,P_{1},H,P_{2}$, respectively (Fig. 7a). Under this model, the probabilities of gene trees and site patterns are both given by a mixture over the two binary species trees $S_{1}$ and $S_{2}$ (called *parental species trees*), with mixing probabilities φ and 1−φ (Fig. 7b and c). Given species tree $S_{1}$, the matching pattern iijj has a larger probability (say, a) than the other two mismatching patterns (each with probability b, say, with b<a). Given species tree $S_{2}$, the matching pattern ijji has probability a while the two mismatching patterns have b each. The symmetry assumptions ($\tau_{S}=\tau_{T}$ and $\theta_{S}=\theta_{T}$) ensure that a,b for tree $S_{1}$ are equal to a,b for $S_{2}$. By averaging over the two species trees, the site-pattern probabilities under the hybridization model are given as


$$\begin{array}{l} \begin{array}{lll} p_{iijj}=\varphi a+\left(1-\varphi \right)b & \; & \\ p_{ijij}=\varphi b+\left(1-\varphi \right)b=b & \; & \\ p_{ijji}=\varphi b+\left(1-\varphi \right)a. & \; & \end{array} \end{array}$$ (8)

Setting those probabilities to the observed frequencies ($\hat{p}$) and eliminating a and b from the system of equations gives the estimate


$$\begin{array}{l} \hat{\varphi }=\frac{{\hat{p}}_{iijj}-{\hat{p}}_{ijij}}{{\hat{p}}_{iijj}-2{\hat{p}}_{ijij}+{\hat{p}}_{ijji}}, \end{array}$$ (9)

This is equation (3) by Blischak *et al.* (2018), although the derivation here is simpler than that of Kubatko and Chifman (2019). Note that the theory works if $\tau_{S}=\tau_{T}>\tau_{X}$ and $\theta_{S}=\theta_{T}$, so that the method may be used under model A of Flouri *et al.* (2020, Fig. 1) with the symmetry assumption. The null hypothesis of no hybridization/introgression ($H_{0}:\varphi =0$) can be tested by applying a normal approximation to the site-pattern counts (Kubatko and Chifman, 2019).

To see which of the two assumptions ($\tau_{S}=\tau_{T}$ and $\theta_{S}=\theta_{T}$) has more impact, note that a change in τ is comparable with the same amount of change in 2/*θ*. Coalescent may occur in population RS (if the H sequence takes the left parental path in the model of Fig. 7a), at the rate 2/*θ*_*S*_ over time period $\tau_{R}-\tau_{S}$, and it may occur in population RT (if the H sequence takes the right parental path), at the rate 2/*θ*_*T*_ over time period $\tau_{R}-\tau_{T}$. If $2\left(\tau_{R}-\tau_{S}\right)/\theta_{S}=2\left(\tau_{R}-\tau_{T}\right)/\theta_{T}$, the probability of coalescent (given that two sequences enter populations S or T) will be the same in the two populations. However, the probabilities of the site patterns depend on the time of coalescent as well as its occurrence. Thus for equation (9) to be valid, both the rates and the times have to be identical: $\tau_{S}=\tau_{T}$ and $\theta_{S}=\theta_{T}$.

Note that HYDE or the D-statistic cannot be used to infer gene flow between sister lineages. One might think that HYDE or D could be applicable if two sequences were sampled from the recipient lineage to form a quartet. However this is not the case. With ancient introgression, the two sequences from the same lineage are interchangeable and have the same average genomic distance to the outgroup sequence. Suppose $P_{1}$ and H in Fig. 7a are two sequences from the same lineage. Then site patterns iijj and ijij will have the same probability even if φ>0.

### Simulations to Examine the Performance of HYDE

**Figure 8.**
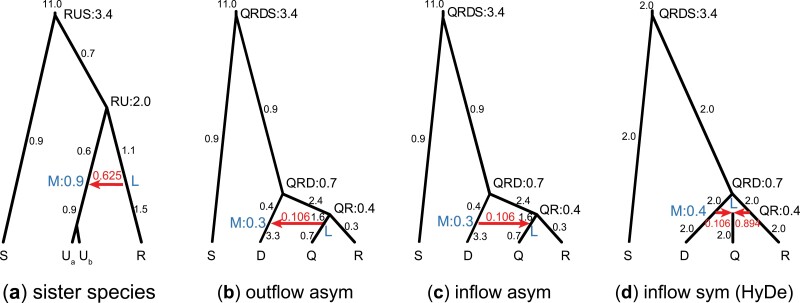
Introgression models (species trees with introgression) used for simulating data to evaluate the performance of HYDE and BPP. (a) Species tree for three species (R, U, and S) with R→U introgression at the rate of φ=0.625, and with S to be the outgroup, based on BPP estimates from the *Tamias* data (Fig. 4b, Table S3). Population sizes (θ) are next to the branches and species divergence times (τ) are next to the nodes. Two sequences are sampled from species U. When the data are analyzed using HYDE, either Ua or Ub is specified as the hybrid lineage. (b) Outflow model for three species (D, Q, R), with S to be the outgroup, with introgression from Q to D at the rate φ=0.106 (Table S3). (c) Inflow asymmetrical model for three species, with asymmetrical divergence times and population sizes. (d) Inflow symmetrical model for three species, with $\tau_{M}=\tau_{QR}$ and $\theta_{M}=\theta_{QR}$ (see Fig. 7a). Note that only model (d) matches the assumption of HYDE.

**Figure 9.**
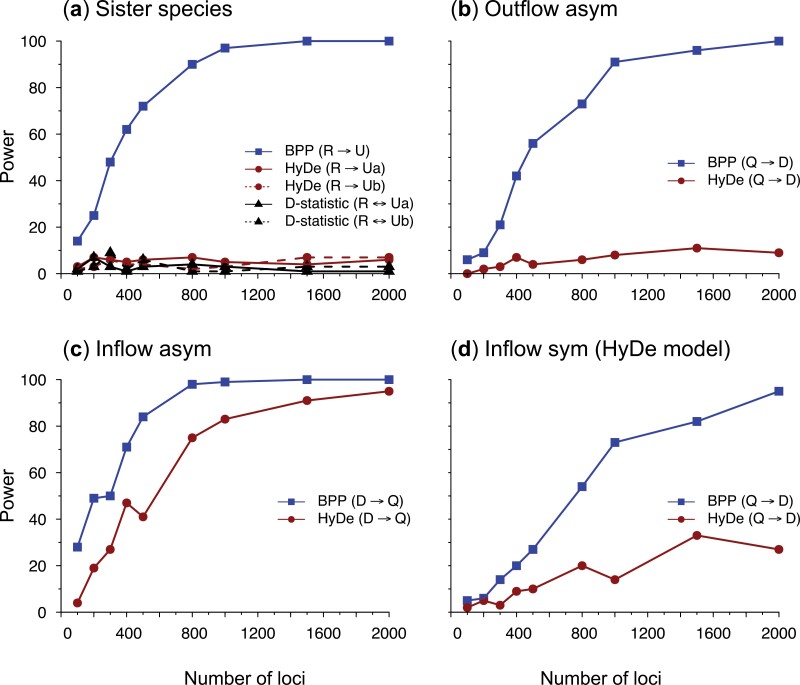
Power of detecting gene flow by HYDE and BPP in 100 replicate datasets simulated under the models of Fig. 8.

Our examination of assumptions underlying HYDE suggests that HYDE may not be suitable for testing gene flow in the *Tamias* data. The strongest introgression in the *Tamias* data detected using BPP was between sister species, with $\varphi_{QIRCD\to U}=0.625$ (Fig. 4b). This is unidentifiable by HYDE. The next introgression involved outflow with $\varphi_{QI\to D}=0.106$, whereas HYDE assumes inflow. The third introgression was again between sister species, with $\varphi_{Q\to I}=0.050$. To verify those expectations and to explore the performance of HYDE and BPP under different scenarios of gene flow, we conducted simulations using four different model settings (Fig. 8a–d), based on parameter estimates obtained from the *Tamias* data (Fig. 4b, Table S3). Gene trees and sequence alignments at multiple loci were generated using the simulate option of BPP. HYDE analysis was conducted using PAUP (Swofford, 2003). The data were also analyzed using BPP. The results are summarized in Fig. 9.

Model a (Fig. 8a) assumes gene flow between sister lineages, based on the introgression event from QIRCD → U in the *Tamias* data (Fig. 4b). It was suggested that by including multiple sequences from the recipient lineage, HYDE or the D-statistic might be used to detect gene flow between sister lineages. We used species R and U, with introgression rate $\varphi_{R\to U}=0.625$, including two sequences (Ua and Ub) from the recipient species U, while S was used as the outgroup. The divergence times (τ) and population sizes (θ) were based on the real data (Table S3). When multiple branches in the full tree (Fig. 4b) were merged into one branch in the tree of Fig. 8a, θ for the merged branch was calculated as a weighted average, with the branch lengths as weights. As our objective in this case was to confirm the lack of power of HYDE (and the D-statistic), we simulated large datasets, each with L=8000 loci. The sequence length was 500 sites, and the number of replicates was 100. When the data were analyzed using HYDE and the D-statistic, the quartet tree (((Ua, Ub), R), S) was used, with Ua or Ub labeled the “hybrid” lineage. The same data were analyzed using BPP under the MSci model with three species (Fig. 8a).

As expected, HYDE and the D-statistic had no power to detect gene flow between sister lineages: indeed, the power of HYDE and D was not higher than the significant level (Fig. 9, Table S4). Note that a test that ignores data and produces 5% positives at random will have 5% of power. Also HYDE did not produce reliable estimates of φ; in about half of the datasets, the estimate was outside the range (0,1).

Model b (Fig. 8b) was based on the next strongest introgression in the *Tamias* data, with $\varphi_{QI\to D}=0.106$ (Fig. 4b). We used species D, Q, R, with S as the outgroup. This is a case of outflow, when gene flow from an ingroup species Q to a more distant species D. Our examination of the assumptions made by HYDE suggests that HYDE can be used to detect inflow, but not outflow. We generated datasets of various sizes with L=500,2000, or 8000 loci. The other settings were the same as for model a. When the data were analyzed using HYDE, *Q* was designated the “hybrid” lineage while *R* and *D* were the two parents. HYDE performed poorly (Fig. 9b), with very low power and frequent invalid estimates of φ (Table S5).

Model c (Fig. 8c) was the same as model b but the direction of gene flow was reversed. The model was then a case of inflow, as assumed by HYDE. However, species divergence times and population sizes did not satisfy the symmetry requirements of HYDE (in other words, $\tau_{M}\neq \tau_{QR}$ and $\theta_{M}\neq \theta_{QR}$). In this case, HYDE had considerable power in detecting gene flow (Fig. 9c). However, the estimates of φ by HYDE involved large biases, apparently converging to ≈0.32 when the true value was 0.106 (Table S5). This positive bias is apparently because coalescent occurs at a higher rate or over longer time period on the M branch than on the QR branch in Fig. 8c, with $(\tau_{QRD}-\tau_{M})/\theta_{M}>(\tau_{QRD}-\tau_{QR})/\theta_{QR}$. In the opposite case, the bias should be negative.

Model d (Fig. 8d) was the same as model c with inflow but in addition we enforced the symmetry assumptions, so that species Q was a hybrid species formed by hybridization between D and R. This is the hybrid-speciation model assumed by HYDE, and the method performed well (Fig. 9d). Its power was lower than that for BPP, as expected from statistical theory, but improved with the increase of data, rising from 10% at L=500 loci to 90% at 8000 loci. The parameter estimate appeared to be consistent, converging to the correct value (0.106) when the number of loci increased, and there were not many invalid estimates (Table S5). Those results are consistent with previous simulations, which evaluated the performance of HYDE when all its assumptions were met and found the method to perform well (Blischak *et al.*, 2018; Flouri *et al.*, 2020).

In summary, our simulations suggest that it is important to apply HYDE to detect the correct mode of gene flow (that is, gene flow between nonsister lineages, and inflow instead of outflow) (Fig. 8d). Furthermore, the symmetry assumptions are important for HYDE to produce reliable estimates of introgression probability. When all model assumptions are met, HYDE performed well. However, HYDE had no power to detect gene flow between sister lineages, and very low power to detect outflow.

**Figure 10.**
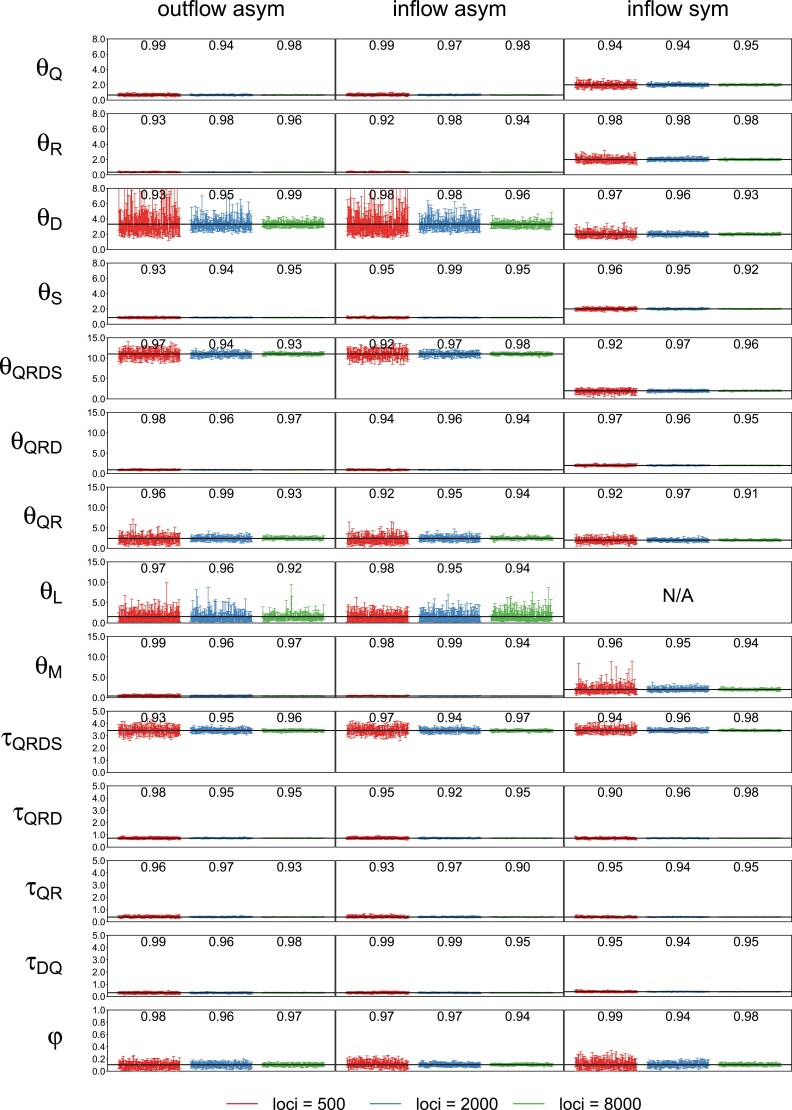
Posterior means and 95% HPD CIs for parameters in the three introgression models of Fig. 8: (b) outflow asym, (c) inflow asym and (d) inflow sym (HYDE model), in BPP analyses of 100 replicate datasets, each with 500, 2000, or 8000 loci. Note that in model (d) inflow sym, all populations had the same size (θ) although separate θ parameters were estimated for different populations when the data were analyzed using BPP. Parameters τ and θ are multiplied by 10^3^. The number above the CI bars is the coverage or the probability that the CI includes the true value.

In all four models (Fig. 8a–d), the Bayesian test using BPP had good power (Fig. 9, Tables S4 and S5). Furthermore, the posterior means and 95% HPD CIs for parameters in the introgression models b-d were well behaved (Fig. 10). While HYDE can estimate only two parameters from the site-pattern counts (the internal branch length in coalescent units on the species tree and the introgression probability), the BPP analysis of the same data estimates all parameters in the model. The species divergence/introgression times were all well estimated with small CIs (Fig. 10). The introgression probability was accurately estimated with narrow CIs when ≥500 loci were used. Population size parameters for short branches were poorly estimated due to lack of coalescent events in those populations.

We also examined the false positive rate (type I error rate) of the HYDE and Bayesian tests, by simulating data using the inflow-asym (Fig. 8c) and inflow-sym (Fig. 8d) models but with φ=0 fixed so that there was no introgression in the true model. The results are summarized in Table 3. Under the inflow-asym model, HYDE had higher false positive rate than the nominal significant level. For example, at the 5% significance level, the false positive rate was 7%, 13%, and 7% in datasets of 500, 2000, and 8000 loci, respectively. The high rate may be explained by the violation of the symmetry assumptions for HYDE. Under the inflow-sym model (or the HYDE model), the rate was 3%, 2%, and 3%, all within the allowed 5% (Table 3). Thus HYDE performed well when its assumptions were met and had elevated false positives when the assumptions were violated. In all settings, the false positive rate of the Bayesian test was estimated to be ∼0% . This is consistent with the expectation that the Bayesian test may be more conservative (with lower false positive rate and lower power) than the LRT (see discussions later).

**Table 3:** False positive rate of bpp and HYDE tests and average estimates of introgression probability in 100 simulated replicates

	bpp			HYDE			
	Error rate	Error rate		Error rate	Error rate		Proportion of invalid estimates
# loci	(α=1% )	(α=5% )	$\hat{\varphi }\pm$ SD	(α=1% )	(α=5% )	$\hat{\varphi }\pm$ SD	
Inflow asym (Fig. 8c)							
500	0%	0%	0.019±0.011	1%	7%	0.140±0.108	52%
2000	0%	0%	0.009±0.004	5%	13%	0.094±0.061	52%
8000	0%	0%	0.004±0.002	2%	7%	0.038±0.032	51%
Inflow sym (Fig. 8d, HyDe model)							
500	0%	0%	0.032±0.016	0%	3%	0.064±0.048	49%
2000	0%	0%	0.014±0.006	1%	2%	0.039±0.029	55%
8000	0%	0%	0.006±0.003	0%	3%	0.022±0.016	49%

Note: Data were simulated using the species trees of Fig. 8c, d but with φ=0.

Finally, to assess the information content in datasets of the size of the *Tamias* data, we used parameter estimates from the full dataset (Fig. 4b, Table S3) to simulate two datasets of the same size as the original, with 5, 5, 9, 10, 11, 11, 3 unphased sequences per locus for species R, C, I, U, Q, D, and S, respectively. The sequence length was 200 sites. We analyzed the datasets under the same MSci model of Fig. 4b using BPP to estimate all parameters. The estimates from the two datasets were similar, so we present those from one of them in Table S3. At this data size, BPP achieved relatively good precision and accuracy. The posterior means were close to the true values, and the CIs were also similar to those calculated from the real data. Similarly to analyses of the real data, divergence times and population sizes for modern species were well estimated, but ancestral population sizes, in particular those for populations of short time duration, were more poorly estimated.

## Discussion

### Criteria for Testing Gene Flow

Hypothesis testing or model selection involves arbitrariness, and classical hypothesis testing and Bayesian model selection applied to the same data may produce strongly opposed conclusions, a situation known as Jeffreys’s paradox (Jeffreys, 1939; Lindley, 1957). Furthermore, Bayesian model selection is known to be sensitive to priors on model parameters, especially on parameters that are not shared between the models under comparison. See Yang (2014, pp. 194–7) for a discussion of those issues. Here we review different strategies for testing, using as example a simple problem of testing the null hypothesis $H_{0}:\mu =0$ against the alternative $H_{1}:\mu \neq 0$, using a data sample, , from the normal distribution N(μ,1). We assume that a false positive error (of falsely rejecting $H_{0}$ when it is true) is more serious than a false negative error (of failing to reject $H_{0}$ when it is false). The data can be summarized as the sample mean $\bar{x}$, with the likelihood given by $\bar{x}∼N\left(0,1/n\right)$ under $H_{0}$ and $\bar{x}∼N\left(\mu ,1/n\right)$ under $H_{1}$. Let $\phi \left(x;\mu ,\sigma^{2}\right)$ be the probability density function (PDF) for $N\left(\mu ,\sigma^{2}\right)$ and Φ(⋅) be the CDF for N(0,1).

In hypothesis testing, the *P*-value can be calculated from the fact that under $H_{0}$, $\sqrt{n}|\bar{x}|∼N\left(0,1\right)$ or $n|\bar{x}{\;\;\;|\;\;\;}^{2}∼\chi_{1}^{2}$. At the α=5%  significance level, we reject $H_{0}$ if


$$\begin{array}{l} 2\Delta \ell =2\log\frac{\phi (\bar{x};\bar{x},1/n)}{\phi (\bar{x};0,1/n)}=n|\bar{x}{|}^{2}>\chi_{1,5\%}^{2}=3.84. \end{array}$$ (10)

Alternatively one may consider this as an estimation problem and construct a confidence interval (CI) for μ and reject $H_{0}$ if the CI excludes the null value 0. This is equivalent to the LRT.

In a Bayesian analysis, we consider two approaches. The first is to examine whether the posterior 95% credibility interval (CI) for μ under $H_{1}$ excludes the null value 0. We assign the prior $\mu ∼N\left(0,\sigma_{0}^{2}\right)$ under $H_{1}$. The posterior is then $\mu |x∼N(\mu_{1},\sigma_{1}^{2})$, with mean $\mu_{1}=n\bar{x}/(n+1/\sigma_{0}^{2})$ and precision $1/\sigma_{1}^{2}=n+1/\sigma_{0}^{2}$. Here the reciprocal of variance is known as precision. The sample precision is n and the prior precision is $1/\sigma_{0}^{2}$ , while the posterior precision is the sum of the two. The 95% CI for μ is given as $\mu_{1}\pm 1.96\sigma_{1}$ so that the CI excludes 0 (in which case we reject $H_{0}$) if $|\mu_{1}|>1.96\sigma_{1}$, or if


$$\begin{array}{l} n|\bar{x}{|}^{2}>3.84\left[1+1/(n\sigma_{0}^{2})\right]. \end{array}$$ (11)

The second approach is to use the Bayes factor to compare the null and alternative hypotheses (e.g., Yang, 2006, eq. 5.21).


$$\begin{array}{l} \begin{array}{ll} & B_{10}=\frac{P(\bar{x}|H_{1})}{P(\bar{x}|H_{0})}=\frac{\phi \left(\bar{x};0,\frac{1}{n}+\sigma_{0}^{2}\right)}{\phi \left(\bar{x};0,\frac{1}{n}\right)}\\ & \;\;\;=\frac{1}{\sqrt{1+n\sigma_{0}^{2}}}\cdot \exp\left\{\frac{n{\bar{x}}^{2}}{2\left[1+1/(n\sigma_{0}^{2})\right]}\right\}, \end{array} \end{array}$$ (12)

The Bayes factor is closely related to (and ‘calibrated’ using) the posterior model probability. If the two models are assigned equal prior probabilities ($\pi_{0}=\pi_{1}=1/2$), the posterior model probability is


$$\begin{array}{l} P\left(H_{1}\;|\;x\right)=\frac{B_{10}}{1+B_{10}}, \end{array}$$ (13)

so that a 95% cutoff on $P\left(H_{1}\;|\;x\right)$ corresponds to $B_{10}=19$,and $H_{0}$ is rejected based on the Bayes factor if and only if


$$\begin{array}{l} n|\bar{x}{|}^{2}>\log\left\{19\sqrt{1+n\sigma_{0}^{2}}\right\}\times 2\left[1+1/(n\sigma_{0}^{2})\right]. \end{array}$$ (14)

While the LRT (equation (10)) depends on $\sqrt{n}|\bar{x}|$ only, both the posterior CI (equation (11)) and the Bayes factor (equation (14)) depend in addition on $n\sigma_{0}^{2}$. Note that the three criteria (equations (10), (11), and (14)) have the ordering


$$\begin{array}{l} \begin{array}{ll} & 3.84<3.84\left[1+1/(n\sigma_{0}^{2})\right]\;\\ & \;<\log\left\{19\sqrt{1+n\sigma_{0}^{2}}\right\}\times 2\left[1+1/(n\sigma_{0}^{2})\right]. \end{array} \end{array}$$ (15)

Thus the LRT has more power and higher false positive rate than the posterior CI while the Bayesian test based on the Bayes factor is the most conservative. The result reflects the general perception that the LRT tends to reject the null hypothesis and favor parameter-rich models too often, especially in large datasets. Note that if $H_{0}$ is true, the false positive rate of the LRT stays at 5%  when the sample size n→∞, whereas in the Bayesian analysis, the true model $H_{0}$ will dominate, with $P\left(H_{0}\;|\;x\right)\to 1$ and $B_{10}\to 0$ when n→∞.

Example calculations are given in Table 4 for two datasets with $\sqrt{n}|\bar{x}|=1.96$ or 2.58 and n=100. In both datasets, $H_{0}$ is rejected by the LRT (at the 5% and 1% levels, respectively), but the Bayes factor and the posterior model probabilities favor $H_{0}$ over $H_{1}$, with $B_{10}<1$ and $P\left(H_{1}\;|\;x\right)<1/2$.

**Table 4:** LRT and Bayesian tests in the normal example in two datasets

Data	LRT	Bayesian test		
$\sqrt{n}|\bar{x}|$	*P*-value	Prior	$B_{10}$	$P\left(H_{1}\;|\;x\right)$
1.96	0.05	$\sigma_{0}=1$	0.359	0.264
1.96	0.05	$\sigma_{0}=2$	0.262	0.208
1.96	0.05	$\sigma_{0}=10$	0.120	0.107
2.58	0.01	$\sigma_{0}=1$	0.408	0.290
2.58	0.01	$\sigma_{0}=2$	0.300	0.230
2.58	0.01	$\sigma_{0}=10$	0.138	0.122

Note: The Bayes factor $B_{10}$ is calculated assuming data size n=100 in equation (12), while the posterior model probability is given by equation (13). Note that the *P*-value for the LRT is 5% (or 1%) in the dataset with $\sqrt{n}|\bar{x}|=1.96$ (or 2.58).

This analysis suggests that the difference in power between HYDE and BPP are due to the inefficient use of information in the data by HYDE, not to the different statistical philosophies. An LRT for testing introgression applied to the multilocus sequence alignments may be expected to have more power (and higher false positive rate) than the Bayesian test based on the Bayes factor.

### The Power of Heuristic and Likelihood Methods to Detect Introgression

When applied to the *Tamias* dataset, HYDE and BPP produced opposite conclusions concerning gene flow. Our examination of the model assumptions for HYDE and our simulations suggest that this is because gene flow with the strongest signal in the *Tamias* group, either between sister species or involving outflow, may be of the wrong type or in the wrong direction for HYDE. Here we review and summarize the major issues with HYDE.

First, both HYDE and the D-statistic pool sites across loci when counting site patterns, so that the site-pattern counts are genome-wide averages. Cross-species gene flow creates genealogical variation across the genome, with the probabilistic distribution of the gene trees and coalescent times specified by parameters in the MSC model with gene flow, such as species divergence times, population sizes, and rates of gene flow (Barton, 2006; Lohse and Frantz, 2014). As a result, there is important information concerning gene flow in the variance of site-pattern counts among loci, but this information is ignored by those methods. In other words, sites at the same locus share the genealogical history under the assumption of no within-locus recombination (see Zhu *et al.*, 2022 for an evaluation of the impact of this assumption on MSC-based analyses), and their differences reflect the stochastic fluctuation of the mutation process. Sites at different loci in addition may have different genealogical histories, reflecting the stochastic nature of the process of coalescent and introgression. When sites are pooled across loci, those two sources of variation are confounded, leading to loss of information (Shi and Yang, 2018; Zhu and Yang, 2021). As a consequence, certain forms of introgression, such as introgression between sister lineages, are unidentifiable by D or HYDE, while estimation of introgression rates between nonsister species suffers from larger variances (Jiao *et al.*, 2021).

Second, HYDE makes restrictive assumptions about gene flow. The underlying model is one of hybrid speciation with identical population sizes or equivalently the inflow model with symmetrical species divergence times and population sizes (Fig. 7a, with $\tau_{S}=\tau_{T}$ and $\theta_{S}=\theta_{T}$) (Blischak *et al.*, 2018; Kubatko and Chifman, 2019). Our simulation suggests that HYDE can indeed infer gene flow/hybridization and produce reliable estimates of introgression probability under this model (Fig. 9d; Table S5; see also Blischak *et al.*, 2018; Flouri *et al.*, 2020). However, introgression in the wrong direction or violation of the symmetry assumptions may lead to loss of power and biased or invalid estimates by HYDE (Fig. 9b, c, Table S5).

Third, the approaches taken by HYDE to accommodate multiple samples per species and heterozygote sites in diploid genomes may be problematic. When multiple samples are available in the species quartet, HYDE counts site patterns in all combinations of the quartet. Let the numbers of sequences for species $O,P_{1},H,P_{2}$ be $n_{O},n_{1},n_{H},n_{2}$. There are then $n_{O}\times n_{1}\times n_{H}\times n_{2}$ combinations in which one sequence is sampled per species, and HYDE counts site patterns in all of them (Blischak *et al.*, 2018). This ignores the lack of independence among the quartets and exaggerates the sample size. At the same time, multiple samples from the same species are never compared with each other, which should provide important information about the population size for that species. In a likelihood method such as BPP, all sequences at the same locus, both from the same species and from different species, are related through a gene tree, and genealogical information at the locus is used.

Similarly heterozygote sites are not treated properly in HYDE. If the site pattern is AGRG, with R representing an A/G heterozygote, HYDE adds 0.5 each to the site patterns ijjj (for AGGG) and ijij (for AGAG) (Blischak *et al.*, 2018), in effect treating R as an unknown nucleotide that is either A or G whereas correctly it means a heterozygote (both A and G). The proportion of heterozygotes in each diploid genome should be informative about θ for that population, but such information is not used by HYDE. In BPP, heterozygote sites are resolved into their underlying nucleotides using an analytical integration algorithm (so that R means both A and G, say), with the uncertainty in the genotypic phase of multiple heterozygous sites in a diploid sequence accommodated by averaging over all possible heterozygote phase resolutions, weighting them according to their likelihoods based on the sequence alignment at the locus (Gronau *et al.*, 2011; Flouri *et al.*, 2018). Simulations suggest that this approach has nearly identical statistical performance to using fully phased haploid genomic sequences (Gronau *et al.*, 2011; Huang *et al.*, 2022).

In this paper, we have focused on the heuristic method HYDE and the likelihood method BPP, as they have been used to analyze the *Tamias* data. By choosing parameter values to be representative of the *Tamias* data, our simulation has evaluated a tiny portion of the parameter space and does not constitute a systematic evaluation of the performance of HYDE. The strengths and weaknesses of heuristic and likelihood methods for inference under models of gene flow were discussed by Degnan (2018) and Jiao *et al.* (2021), but a comprehensive comparative study has not yet been conducted. For estimation of the species phylogeny under the MSC without gene flow (Zhu and Yang, 2021, Fig. 3) demonstrated a dramatic information loss resulting from pooling sites across loci in the site-pattern based methods (also known as coalescent-aware concatenation methods), and from the failure to use information in coalescent times or gene-tree branch lengths in the two-step methods (which infer the gene trees and then treat them as data to infer the species tree). Both the site pattern-based and the two-step methods are used to infer gene flow and to estimate the introgression probability (e.g., HYDE and the D-statistic in the first category and SNAQ in the second) and similar information loss may be expected. A detailed analysis of the performance of heuristic methods in comparison with likelihood methods will be interesting. Currently, the gap between the heuristic and likelihood methods appears to be a large one. Heuristic methods are orders-of-magnitude more efficient computationally and can be applied to much larger datasets, whereas likelihood methods have far better statistical properties, being able to identify and estimate all parameters in the model. There are great opportunities for improving both the statistical performance of heuristic methods and the computational efficiency of likelihood methods (including the mixing efficiency of MCMC algorithms).

### Introgression in *T. quadrivittatus* Chipmunks

The joint introgression model for the *T. quadrivittatus* group (Fig. 4b) was constructed using a stepwise approach that iteratively adds introgression events to the binary species tree. We note several limitations with this approach. First the approach assumes the availability of a stable binary species tree, and may not be feasible if the species tree is large and highly uncertain, possibly influenced by introgression events (Leaché *et al.*, 2014). The *Tamias* dataset analyzed here includes only six species, and the first stage of our procedure (i.e., the separate analysis) involved 16 possible introgression events, so that the computation was feasible. Second, the approach is not an exhaustive search in the space of introgression models and may miss certain introgression events. Note that introgression events not selected in the first stage of the procedure will not be incorporated in the final joint introgression model. In our analysis of the *Tamias* data, we considered introgressions between contemporary species, mostly based on phylogenetic analyses of the mitochondrial genome (Sarver *et al.*, 2017), and moved certain events to older ancestral branches when the estimated introgression time coincided with the species divergence time. We did not evaluate introgressions involving ancestral branches systematically. Furthermore, the criterion based on the Bayes factor used in our test is a stringent one, and the dataset of 1060 loci is relatively small. All those factors suggest that we cannot rule out the possibility that we may have missed some introgression events; in other words, our analysis may suffer from false-negative errors. In contrast, the three introgression events identified in our analysis (Fig. 4b) appear to be robust and are unlikely to be false positives (Fig. 5, Table S2). We conclude that there is strong and robust evidence that gene flow has affected the nuclear genome in the *T. quadrivittatus* group of chipmunks.

Given the extensive mitochondrial introgression in the *Tamias* group (Sullivan *et al.*, 2014; Sarver et al., 2017, 2021), introgression affecting the nuclear genome was expected, and the failure to detect any significant evidence for it in the HYDE analysis was surprising (Sarver *et al.*, 2021). Sarver *et al.* (2021) discussed the evidence for cytonuclear discordance in the pattern of introgression (Bonnet *et al.*, 2017; McElroy *et al.*, 2020; Sarver *et al.*, 2021), as well as possible roles of purifying selection affecting the coding genes or exons that make up the nuclear dataset being analyzed. Our results suggest a simpler explanation, that gene flow in the *Tamias* group is of a wrong type or in the wrong direction, undetectable by HYDE.

Our analyses suggest that species involved in excessive mitochondrial introgression tend to be those involved in nuclear introgression as well. *T. dorsalis* was noted to be a universal recipient of mtDNA from other species (Sullivan *et al.*, 2014; Sarver *et al.*, 2017). Consistent with this, our separate analysis (Table 2) identified three introgression events into *T. dorsalis* with φ>5%  as well as one event with *T. dorsalis* to be the donor species, even though some of those events become non-significant after introgression involving older ancestors was incorporated in the model. It will be interesting to use expanded datasets to examine whether this is due to a lack of power to detect gene flow or a genuine lack of gene flow.

It will be very useful to generate more genomic data, especially the noncoding parts of the nuclear genome, including more species from the genus, to provide more power for detecting gene flow and estimating introgression rates. It will also be interesting to examine whether the noncoding and coding regions of the genome give consistent signals concerning species divergences and cross-species gene flow, and to examine how the effective rate of gene flow vary among chromosomes or across genomic regions. In a few genomic analyses, coding and noncoding parts of the genome were found to produce highly consistent results, with nearly proportional estimates of divergence times (τ) and population sizes (θ), and with very similar estimates of introgression rates (Shi and Yang, 2018; Thawornwattana et al., 2018, 2022). One can also examine the posterior distribution of the gene trees to identify loci or genomic segments that are most likely to have been transferred across species boundaries, and to correlate with the functions of genes residing in or tightly linked to the segments.

## Supplementary Material

Data available from the Dryad Digital Repository: https://doi.org/10.5061/dryad.fxpnvx0t9.

## Appendix: Bayes Factor as the Savage-Dickey Density Ratio in Comparison of Nested Models

Consider the comparison of the null model $H_{0}:\phi =\phi_{0}$ against the alternative model $H_{1}:\phi \neq \phi_{0}$, and suppose that both models have common (nuisance) parameters λ, while parameters ξ in $H_{1}$ become unidentifiable when $\phi =\phi_{0}$. The parameter vector is λ for $H_{0}$ and (ϕ,λ,ξ) for $H_{1}$. Given data x, let the likelihood be $L_{0}\left(\lambda \right)$ under $H_{0}$ and L(ϕ,λ,ξ)=p(x | ϕ,λ,ξ) under $H_{1}$, with $L\left(\phi_{0},\lambda ,\xi \right)=L_{0}\left(\lambda \right)$ as the two models are nested. Let the prior be $\pi_{0}\left(\lambda \right)$ under $H_{0}$ and π(ϕ,λ,ξ)=π(ϕ)π(λ | ϕ)π(ξ | ϕ,λ) under $H_{1}$. Under the assumption that the priors on the common parameters (λ) agree between the two models, $\pi \left(\lambda \;|\;\phi_{0}\right)=\pi_{0}\left(\lambda \right)$ (equation (2)), the Bayes factor $B_{10}$ in support of $H_{1}$ over $H_{0}$ (equation (1)) can be expressed as the ratio of the prior and posterior densities for ϕ in $H_{1}$, both evaluated at the null value $\phi_{0}$: that is, $B_{10}=\pi (\phi_{0})/\pi (\phi_{0}\;|\;x)$ (equation (3)).

### PROOF

Rewrite the prior $\pi_{0}\;\left(\lambda \right)$ and likelihood $L_{0}\;\left(\lambda \right)$ under $H_{0}$ as probability densities under $H_{1}$. We have

$$\begin{array}{l} \begin{array}{ll} & B_{10}=\frac{m}{\int \pi_{0}(\lambda )L_{0}(\lambda )d\lambda }\\ & =\frac{m}{\int \pi (\lambda |\phi_{0})L_{0}(\lambda )d\lambda }\\ & =\frac{m}{\int \int \frac{\pi (\phi_{0},\lambda ,\xi )}{\pi (\phi_{0})}L(\phi_{0},\lambda ,\xi )d\xi d\lambda }\;\\ & =\frac{\pi (\phi_{0})}{\int \int \frac{1}{m}\pi (\phi_{0},\lambda ,\xi )L(\phi_{0},\lambda ,\xi )d\xi d\lambda }\\ & =\frac{\pi (\phi_{0})}{\iint \pi (\phi_{0},\lambda ,\xi |x)d\xi d\lambda }\;\\ & =\frac{\pi (\phi_{0})}{\pi (\phi_{0}|x)}. \end{array} \end{array}$$
(A1)

The proof above is more general than that given by Dickey (1971), which does not deal with the unidentifiability of ξ. Thus equation (3) holds even if there exist nuisance parameters (λ) in both models, if the null values ($\phi_{0}$) are at the boundary of the parameter space in $H_{1}$, and if some parameters in $H_{1}$ (ξ) become unidentifiable when the parameters of interest take the null values (when $\phi =\phi_{0}$).

## References

[CIT0001] Arnold B.J. , Lahner B., DaCosta J.M., Weisman C.M., Hollister J.D., Salt D.E., Bomblies K., Yant L. 2016. Borrowed alleles and convergence in serpentine adaptation. Proc. Natl. Acad. Sci. USA 113(29):8320–8325. 27357660 10.1073/pnas.1600405113 PMC4961121

[CIT0002] Barton N.H. 2006. Evolutionary biology: how did the human species form? Curr. Biol. 16:R647–R650. 16920616 10.1016/j.cub.2006.07.032

[CIT0003] Bi K. , Linderoth T., Singhal S., Vanderpool D., Patton J.L., Nielsen R., Moritz C., Good J.M. 2019. Temporal genomic contrasts reveal rapid evolutionary responses in an alpine mammal during recent climate change. PLoS Genet. 15(5):e1008119. 31050681 10.1371/journal.pgen.1008119 PMC6519841

[CIT0004] Blischak P.D. , Chifman J., Wolfe A.D., Kubatko L.S. 2018. HyDe: a Python package for genome-scale hybridization detection. Syst. Biol. 67(5):821–829. 29562307 10.1093/sysbio/syy023 PMC6454532

[CIT0005] Bonnet T. , Leblois R., Rousset F., Crochet P.-A. 2017. A reassessment of explanations for discordant introgressions of mitochondrial and nuclear genomes. Evolution 71(9):2140–2158. 28703292 10.1111/evo.13296

[CIT0006] Brown J.H. 1971. Mechanisms of competitive exclusion between two species of chipmunks. Ecology 52(2):305–311.

[CIT0007] Burgess R. , Yang Z. 2008. Estimation of hominoid ancestral population sizes under Bayesian coalescent models incorporating mutation rate variation and sequencing errors. Mol. Biol. Evol. 25:1979–1994. 18603620 10.1093/molbev/msn148

[CIT0008] Chan Y.C. , Roos C., Inoue-Murayama M., Inoue E., Shih C.C., Pei K.J., Vigilant L. 2013. Inferring the evolutionary histories of divergences in *Hylobates* and *Nomascus* gibbons through multilocus sequence data. BMC Evol. Biol. 13:82. 23586586 10.1186/1471-2148-13-82 PMC3637282

[CIT0009] Chifman J. , Kubatko L. 2014. Quartet inference from SNP data under the coalescent model. Bioinformatics 30(23):3317–3324. 25104814 10.1093/bioinformatics/btu530 PMC4296144

[CIT0010] Dalquen D. , Zhu T., Yang Z. 2017. Maximum likelihood implementation of an isolation-with-migration model for three species. Syst. Biol. 66:379–398. 27486180 10.1093/sysbio/syw063

[CIT0011] Dalquest W.W. , Baskin J., Schultz G. 1996. Fossil mammals from a late miocene (clarendonian) site in beaver county, oklahoma. Contributions in Mammalogy: A Memorial Volume Honoring Dr. J. Knox Jones, Jr. Museum of Texas Tech University: 107–137.

[CIT0012] Degnan J.H. 2018. Modeling hybridization under the network multispecies coalescent. Syst. Biol. 67(5):786–799. 29846734 10.1093/sysbio/syy040 PMC6101600

[CIT0013] Dickey J.M. 1971. The weighted likelihood ratio, linear hypotheses on normal location parameters. Ann. Math. Statist. 42(1):204–223.

[CIT0014] Ellegren H. , Smeds L., Burri R., Olason P.I., Backstrom N., Kawakami T., Kunstner A., Makinen H., Nadachowska-Brzyska K., Qvarnstrom A., Uebbing S., Wolf J.B.W. 2012. The genomic landscape of species divergence in *Ficedula* flycatchers. Nature 491:756–760. 23103876 10.1038/nature11584

[CIT0015] Finger N. , Farleigh K., Bracken J., Leaché A.D., Francois O., Yang Z., Flouri T., Charran T., Jezkova T., Williams D., Blair C. 2022. Genome-scale data reveal deep lineage divergence and a complex demographic history in the texas horned lizard (*Phrynosoma cornutum*) throughout the southwestern and central USA. Genome Biol. Evol 14(1): doi:10.1093/gbe/evab260 PMC8735750 34849831

[CIT0016] Flouri T. , Jiao X., Rannala B., Yang Z. 2018. Species tree inference with BPP using genomic sequences and the multispecies coalescent. Mol. Biol. Evol. 35(10):2585–2593. 30053098 10.1093/molbev/msy147 PMC6188564

[CIT0017] Flouri T. , Jiao X., Rannala B., Yang Z. 2020. A Bayesian implementation of the multispecies coalescent model with introgression for phylogenomic analysis. Mol. Biol. Evol. 37(4):1211–1223. 31825513 10.1093/molbev/msz296 PMC7086182

[CIT0018] Gelman A. , Meng X. 1998. Simulating normalizing constants: From importance sampling to bridge sampling to path sampling. Stat. Sci. 13:163–185.

[CIT0019] Good J.M. , Demboski J.R., Nagorsen D.W., Sullivan J. 2003. Phylogeography and introgressive hybridization: chipmunks (genus *Tamias*) in the northern Rocky Mountains. Evolution 57(8):1900–1916. 14503631 10.1111/j.0014-3820.2003.tb00597.x

[CIT0020] Good J.M. , Hird S., Reid N., Demboski J.R., Steppan S.J., Martin-Nims T.R., Sullivan J. 2008. Ancient hybridization and mitochondrial capture between two species of chipmunks. Mol. Ecol. 17(5):1313–1327. 18302691 10.1111/j.1365-294X.2007.03640.x

[CIT0021] Good J.M. , Sullivan J. 2001. Phylogeography of the red-tailed chipmunk (*Tamias ruficaudus*), a northern Rocky Mountain endemic. Mol. Ecol. 10(11):2683–2695. 11883882 10.1046/j.0962-1083.2001.01397.x

[CIT0022] Green P. 1995. Reversible jump Markov chain Monte Carlo computation and Bayesian model determination. Biometrika 82:711–732.

[CIT0023] Gronau I. , Hubisz M.J., Gulko B., Danko C.G., Siepel A. 2011. Bayesian inference of ancient human demography from individual genome sequences. Nature Genet 43:1031–1034. 21926973 10.1038/ng.937 PMC3245873

[CIT0024] Heled J. , Drummond A.J. 2010. Bayesian inference of species trees from multilocus data. Mol. Biol. Evol. 27:570–580. 19906793 10.1093/molbev/msp274 PMC2822290

[CIT0025] Heller H.C. 1971. Altitudinal zonation of chipmunks (*Eutamias*): interspecific aggression. Ecology 52(2):312–319.

[CIT0026] Hey J. , Chung Y., Sethuraman A., Lachance J., Tishkoff S., Sousa V.C., Wang Y. 2018. Phylogeny estimation by integration over isolation with migration models. Mol. Biol. Evol. 35(11):2805–2818. 30137463 10.1093/molbev/msy162 PMC6231491

[CIT0027] Hird S. , Reid N., Demboski J., Sullivan J. 2010. Introgression at differentially aged hybrid zones in red-tailed chipmunks. Genetica 138(8):869–883. 20623163 10.1007/s10709-010-9470-z

[CIT0028] Huang J. , Bennett J., Flouri T., Yang Z. 2022. Phase resolution of heterozygous sites in diploid genomes is important to phylogenomic analysis under the multispecies coalescent model. Syst. Biol 71:334–352. 34143216 10.1093/sysbio/syab047 PMC8977997

[CIT0029] Huang J. , Flouri T., Yang Z. 2020. A simulation study to examine the information content in phylogenomic datasets under the multispecies coalescent model. Mol. Biol. Evol. 37(11):3211–3224. 32642765 10.1093/molbev/msaa166

[CIT0030] Jeffreys, H. 1939. Theory of Probability. Oxford: Clarendon Press.

[CIT0031] Jiao X. , Flouri T., Yang Z. 2021. Multispecies coalescent and its applications to infer species phylogenies and cross-species gene flow. Nat. Sci. Rev. 8(12). doi:10.1093/nsr/nwab127 PMC8692950 34987842

[CIT0032] Kubatko L.S. , Chifman J. 2019. An invariants-based method for efficient identification of hybrid species from large-scale genomic data. BMC Evol. Biol. 19(1):112. 31146685 10.1186/s12862-019-1439-7 PMC6543680

[CIT0033] Kumar V. , Lammers F., Bidon T., Pfenninger M., Kolter L., Nilsson M.A., Janke A. 2017. The evolutionary history of bears is characterized by gene flow across species. Sci. Rep. 7:46487. 28422140 10.1038/srep46487 PMC5395953

[CIT0034] Lartillot N. , Philippe H. 2006. Computing Bayes factors using thermodynamic integration. Syst. Biol. 55:195–207. 16522570 10.1080/10635150500433722

[CIT0035] Leaché A.D. , Harris R.B., Rannala B., Yang Z. 2014. The influence of gene flow on species tree estimation: a simulation study. Syst. Biol. 63(1):17–30. 23945075 10.1093/sysbio/syt049

[CIT0036] Lindley D. 1957. A statistical paradox. Biometrika 44:187–192.

[CIT0037] Lohse K. , Frantz L.A. 2014. Neandertal admixture in Eurasia confirmed by maximum-likelihood analysis of three genomes. Genetics 196(4):1241–1251. 24532731 10.1534/genetics.114.162396 PMC3982695

[CIT0038] Mallet J. , Besansky N., Hahn M.W. 2016. How reticulated are species? Bioessays 38(2):140–149. 26709836 10.1002/bies.201500149 PMC4813508

[CIT0039] Mao, Y., Economo, E. P., and Satoh, N. 2018. The roles of introgression and climate change in the rise to dominance of *Acropora* corals. Curr. Biol., 28(21): 3373–3382.e5. 30344117 10.1016/j.cub.2018.08.061

[CIT0040] Martin S.H. , Dasmahapatra K.K., Nadeau N.J., Salazar C., Walters J.R., Simpson F., Blaxter M., Manica A., Mallet J., Jiggins C.D. 2013. Genome-wide evidence for speciation with gene flow in Heliconius butterflies. Genome Res. 23(11):1817–1828. 24045163 10.1101/gr.159426.113 PMC3814882

[CIT0041] Martin S.H. , Jiggins C.D. 2017. Interpreting the genomic landscape of introgression. Curr. Opin Genet. Dev. 47:69–74. 28923541 10.1016/j.gde.2017.08.007

[CIT0042] McElroy K. , Black A., Dolman G., Horton P., Pedler L., Campbell C.D., Drew A., Joseph L. 2020. Robbery in progress: Historical museum collections bring to light a mitochondrial capture within a bird species widespread across southern Australia, the copperback quail-thrush *Cinclosoma clarum*. Ecol. Evol. 10(13):6785–6793. 32724551 10.1002/ece3.6403 PMC7381587

[CIT0043] Mirarab S. , Warnow T. 2015. Astral-ii: coalescent-based species tree estimation with many hundreds of taxa and thousands of genes. Bioinformatics 31(12):i44–i52. 26072508 10.1093/bioinformatics/btv234 PMC4765870

[CIT0044] Nielsen R. , Wakeley J. 2001. Distinguishing migration from isolation: a Markov chain Monte Carlo approach. Genetics 158:885–896. 11404349 10.1093/genetics/158.2.885 PMC1461674

[CIT0045] Ogilvie H.A. , Bouckaert R.R., Drummond A.J. 2017. StarBEAST2 brings faster species tree inference and accurate estimates of substitution rates. Mol. Biol. Evol. 34(8):2101–2114. 28431121 10.1093/molbev/msx126 PMC5850801

[CIT0046] Patterson B.D. , Norris R.W. 2016. Towards a uniform nomenclature for ground squirrels: the status of the Holarctic chipmunks. Mammalia 80(3):241–251.

[CIT0047] Patterson B.D. , Thaeler C.S. Jr. 1982. The mammalian baculum: hypotheses on the nature of bacular variability. J. Mammal. 63(1):1–15.

[CIT0048] Patterson N. , Moorjani P., Luo Y., Mallick S., Rohland N., Zhan Y., Genschoreck T., Webster T., Reich D. 2012. Ancient admixture in human history. Genetics 192(3):1065–1093. 22960212 10.1534/genetics.112.145037 PMC3522152

[CIT0049] Payseur B.A. , Rieseberg L.H. 2016. A genomic perspective on hybridization and speciation. Mol. Ecol. 25(11):2337–2360. 26836441 10.1111/mec.13557 PMC4915564

[CIT0050] Rannala B. , Yang Z. 2017. Efficient Bayesian species tree inference under the multispecies coalescent. Syst. Biol. 66:823–842. 28053140 10.1093/sysbio/syw119 PMC8562347

[CIT0051] Reid N. , Demboski J.R., Sullivan J. 2012. Phylogeny estimation of the radiation of western north American chipmunks (*Tamias*) in the face of introgression using reproductive protein genes. Syst. Biol. 61(1):44. 21878471 10.1093/sysbio/syr094 PMC3243737

[CIT0052] Root J.J. , Calisher C.H., Beaty B.J. 2001. Microhabitat partitioning by two chipmunk species (Tamias) in western Colorado. West. N. Am. Naturalist 61:114–118.

[CIT0053] Sarver B.A. , Demboski J.R., Good J.M., Forshee N., Hunter S.S., Sullivan J. 2017. Comparative phylogenomic assessment of mitochondrial introgression among several species of chipmunks (*Tamias*). Genome Biol. Evol 9(1):7–19. 28172670 10.1093/gbe/evw254 PMC5381575

[CIT0054] Sarver B.A.J. , Herrera N.D., Sneddon D., Hunter S.S., Settles M.L., Kronenberg Z., Demboski J.R., Good J.M., Sullivan J. 2021. Diversification, introgression, and rampant cytonuclear discordance in Rocky Mountains chipmunks (Sciuridae: *Tamias*). Syst. Biol. 70(5):908–921. 33410870 10.1093/sysbio/syaa085 PMC8786503

[CIT0055] Self S. , Liang K.-Y. 1987. Asymptotic properties of maximum likelihood estimators and likelihood ratio tests under nonstandard conditions. J. Am. Stat. Assoc. 82:605–610.

[CIT0056] Shi C. , Yang Z. 2018. Coalescent-based analyses of genomic sequence data provide a robust resolution of phylogenetic relationships among major groups of gibbons. Mol. Biol. Evol. 35:159–179. 29087487 10.1093/molbev/msx277 PMC5850733

[CIT0057] Silverman, B. 1986. Density estimation for statistics and data analysis. London: Chapman and Hall.

[CIT0058] Solis-Lemus C. , Ane C. 2016. Inferring phylogenetic networks with maximum pseudolikelihood under incomplete lineage sorting. PLoS Genet. 12(3):e1005896. 26950302 10.1371/journal.pgen.1005896 PMC4780787

[CIT0059] Sullivan J. , Demboski J., Bell K., Hird S., Sarver B., Reid N., Good J. 2014. Divergence with gene flow within the recent chipmunk radiation (*Tamias*). Heredity 113(3):185–194. 24781803 10.1038/hdy.2014.27 PMC4815644

[CIT0060] Swofford, D. L. 2003. PAUP*: Phylogenetic Analysis by Parsimony (*and Other Methods), Version 4. Sinauer Associates, Sanderland, MA.

[CIT0061] Thawornwattana Y. , Dalquen D., Yang Z. 2018. Coalescent analysis of phylogenomic data confidently resolves the species relationships in the *Anopheles gambiae* species complex. Mol. Biol. Evol. 35(10):2512–2527. 30102363 10.1093/molbev/msy158 PMC6188554

[CIT0062] Thawornwattana Y. , Seixas F.A., Mallet J., Yang Z. 2022. Full-likelihood genomic analysis clarifies a complex history of species divergence and introgression: the example of the Erato-Sara group of Heliconius butterflies. Syst. Biol. 71(5):1159–1177. 35169847 10.1093/sysbio/syac009 PMC9366460

[CIT0063] Verdinelli I. , Wasserman L. 1995. Computing Bayes factors using a generalization of the Savage-Dickey density ratio. J. Am. Stat. Assoc. 90(430):614–618.

[CIT0064] Wen D. , Nakhleh L. 2018. Coestimating reticulate phylogenies and gene trees from multilocus sequence data. Syst. Biol. 67(3):439–457. 29088409 10.1093/sysbio/syx085

[CIT0065] White, J. A. 2010. The Baculum in the Chipmunks of Western North America. Good Press.

[CIT0066] Xu B. , Yang Z. 2016. Challenges in species tree estimation under the multispecies coalescent model. Genetics 204(4):1353–1368. 27927902 10.1534/genetics.116.190173 PMC5161269

[CIT0067] Yang, Z. 2006. Computational molecular evolution. Oxford, UK: Oxford University Press.

[CIT0068] Yang, Z. 2014. Molecular evolution: a statistical approach. Oxford: Oxford University Press.

[CIT0069] Yang Z. 2015. The BPP program for species tree estimation and species delimitation. Curr. Zool. 61(5):854–865.

[CIT0070] Yang Z. , Rannala B. 2010. Bayesian species delimitation using multilocus sequence data. Proc. Natl. Acad. Sci. USA. 107:9264–9269. 20439743 10.1073/pnas.0913022107 PMC2889046

[CIT0071] Zhang C. , Ogilvie H.A., Drummond A.J., Stadler T. 2018. Bayesian inference of species networks from multilocus sequence data. Mol. Biol. Evol. 35(2):504–517. 29220490 10.1093/molbev/msx307 PMC5850812

[CIT0072] Zhu T. , Flouri T., Yang Z. 2022. A simulation study to examine the impact of recombination on phylogenomic inferences under the multispecies coalescent model. Mol. Ecol. 31:2814–2829. 35313033 10.1111/mec.16433 PMC9321900

[CIT0073] Zhu T. , Yang Z. 2012. Maximum likelihood implementation of an isolation-with-migration model with three species for testing speciation with gene flow. Mol. Biol. Evol. 29:3131–3142. 22504520 10.1093/molbev/mss118

[CIT0074] Zhu T. , Yang Z. 2021. Complexity of the simplest species tree problem. Mol. Biol. Evol. 39:3993–4009. 10.1093/molbev/msab009 PMC8382899 33492385

